# Single-Cell and Bulk RNASeq Profiling of COVID-19 Patients Reveal Immune and Inflammatory Mechanisms of Infection-Induced Organ Damage

**DOI:** 10.3390/v13122418

**Published:** 2021-12-02

**Authors:** Alexandrea Bass, Yiran Liu, Sivanesan Dakshanamurthy

**Affiliations:** 1 Department of Biochemistry and Molecular Biology, Georgetown University Medical Center, Washington, DC 20057, USA; ab3804@georgetown.edu (A.B.); yl1299@georgetown.edu (Y.L.); 2 Lombardi Comprehensive Cancer Center, Molecular and Experimental Therapeutic Research in Oncology Program, Georgetown University Medical Center, Washington, DC 20057, USA

**Keywords:** single and bulk RNASeq cell profiling and analysis, immune and inflammatory mechanisms of COVID-19, COVID-19 patients gene expression, COVID-19 organ damage

## Abstract

The SARS-CoV-2 virus’s ability to induce hypercytokinemia and cause multiple organ failure makes it imperative to find effective treatments. To understand the mechanism of viral infection and its effects on organ tissues, we analyzed multiple single-cell and bulk RNAseq data from COVID-19 patients’ organ samples. Various levels of severity of infection were accounted for, with comparative analyses between mild, moderate, and severely infected patients. Our analysis uncovered an upregulation of the innate immune response via several inflammatory genes, IL-2, IL-6, IL-8, IL-17A, and NF-κB. Consequently, we found that the upregulation of these downstream effects can lead to organ injury. The downregulated pathways such as eukaryotic initiation factor 2 (eIF2) and eIF4-mediated host translation, were found to lead to an increased viral translation. We also found that the loss of inhibitory peptides can suppress an overactive innate immune response via NF-κB and interleukin-mediated pathways. Investigation of viral-host protein mapping showed that the interaction of viral proteins with host proteins correlated with the down- and upregulation of host pathways such as decreased eIF2-mediated host translation and increased hypertrophy and fibrosis. Inflammation was increased via the stimulation of pro-inflammatory cytokines and suppression of host translation pathways that led to reduced inflammatory inhibitors. Cardiac hypertrophy and organ fibrosis were the results of increased inflammation in organs of severe and critical patients. Finally, we identified potential therapeutic targets for the treatment of COVID-19 and its deleterious effects on organs. Further experimental investigation would conclusively determine the effects of COVID-19 infection on organs other than the lungs and the effectiveness of the proposed therapeutic targets.

## 1. Introduction

Similar to other SARS coronavirus strains, SARS-CoV-2 is an enveloped, positive-sense ssRNA virus that primarily infects lung epithelial cells to cause severe acute respiratory syndrome. It spreads most easily via aerosolized droplets and close contact. Though the lungs are the primary target of SARS-CoV-2 infection, direct infection of non-pulmonary organs such as enterocyte [1] and kidney organoids [2] have been demonstrated in vitro via angiotensin converting enzyme 2 (ACE2) receptors. The high expression of ACE2 receptors in the gastrointestinal tract and, especially, the heart suggest susceptibility of infection. ACE2 degrades angiotensin II (ANG II) to Angiotensin-(1–7), indicating a protective property against inflammation and fibrosis. ACE2 is internalized during the viral entry process and several studies suggest that this reduction in surface ACE2 may lead to an increase in fibrosis and other severe symptoms associated with viral infection [2]. SARS-CoV-2 infections are known to cause hypercytokinemia, an immune dysregulation also known as a cytokine storm, and lymphopenia, the reduction of lymphocytes [3]. Cytokine storm, where pro-inflammatory cytokines overstimulate the immune system to a detrimental extent, leads to multiple organ failure and death. COVID-19’s ability to trigger pro-inflammatory cytokines such as C-reactive protein (CRP), ferritin, lactate dehydrogenase (LDH), D-dimer, IL-6, and IL-2 is known to activate a cytokine storm—an immune overreaction to viral particles that leads to organ damage [4]. A study by Matsuishi et al. suggested that endothelial dysfunction in severe cases induces multiple organ dysfunction in severe COVID-19 patients [5]. They found that virally induced endothelial dysfunction induced acute kidney injury, livery injury leading to thrombosis and coagulopathy, myocardial injury, and a few cases of hemorrhagic stroke [5].

Viral pathogens are known for contributing to cardiovascular disease and COVID-19 is associated with myocardial injury, acute coronary syndromes, cardiac arrythmias, and heart failure. ACE2 expression in the heart is higher than in the lungs, exhibiting extensive ACE2 expression in its pericytes, cardiomyocytes, and in endothelial cells. In a massive autopsy study in China, by Bian et al., myocardia displayed cellular degeneration, scattered necrosis, interstitial edema, and mild infiltration of monocytes, lymphocytes, and neutrophils [6]. Infected cells are damaged and disturb the delicate micro-environment of the myocardium, leading to capillary dysfunction and inducing micro-circulation disorder. Patients with existing cardiovascular disease are more likely to progress to severe conditions and death. In this same study, kidney samples from the deceased were found to have anemic infarct, hyperemia, segmental hyperplasia, and necrosis [6]. Renal interstitial tissues had congestion, mild inflammatory-cell invasion, and fibrous hyperplasia. Single-cell RNA sequencing revealed the expression of ACE2 and transmembrane serine protease 2 (TMPRSS2)—both known COVID-19 receptors—in podocytes and tubule epithelial cells, indicating the possibility for direct infection of the kidney by the virus [7]. Organ cross-talk and cytokine damage are also theorized to induce kidney injury, and more research is required to confirm the mechanisms of kidney injury in COVID-19 infections [7].

Lung tissues express less ACE2 receptors than gastrointestinal cells and heart cells but are nonetheless the primary target for COVID-19. ACE2 expression is highest in type II alveolar cells. The autopsy of deceased patients displayed the catastrophic effect of the virus on the lungs, pulmonary vessels had vasculitis, thrombosis, and thromboembolism [6]. Several studies found that >50% of COVID-19 infected patients show liver abnormalities [8]. Elevated liver enzymes such as aminotransferases, alkaline phosphatase, gamma-glutamyl transferase (GGT), and bilirubin levels have been observed in critical and deceased patients. In severe infection, liver injury is more prevalent than during mild infection—in mild cases, recovery from viral infection is enough to resolve liver injury. Sun et al. suggested five mechanisms of liver injury: immune-mediated damage from inflammatory response to infection, direct damage via ACE2 receptor-mediated infection of hepatic cells, anoxia leading to multiple organ failure, drug-induced damage via antiviral treatments, and exacerbation of existing liver disease [8].

Tayoun et al. developed categorizations of cases into mild, moderate, or severe based on the following criteria [9]. Asymptomatic and mild cases are characterized by non-life-threatening symptoms such as cough and mild fever. Moderate cases include symptoms that require medical attention and hospitalization such as breathlessness and persistent fever. Severe and critical cases have advanced disease and viral pneumonia that require life-support treatment and admission to intensive care units. He et al. found the following symptoms had higher initial prevalence in severe versus mild cases: fever; respiratory symptoms such as cough, dyspnea, expectoration, and hemoptysis; digestive symptoms such as abdominal pain and diarrhea. The following symptoms were not significantly different in mild or severe patients: chest tightness, pharyngalgia, nausea and vomiting, headache, and myalgia [10]. Severe cases have higher mortality than mild cases, due to the increased likelihood of acute respiratory distress syndrome (ARDS), septic shock, metabolic acidosis, and other critical symptoms. The existence of pre-existing conditions is correlated with more severe outcomes for COVID-19 patients; however, more research is needed to confirm if the virus is directly or indirectly interacting with these altered pathways. Nigro et al. found that patients’ metabolic-associated diseases often exhibit worse COVID-19 outcomes [11]. Those with metabolic perturbations, such as diabetes, were not more prone to infection, but were more likely to develop a severe form of infection.

In this study, we performed single-cell and bulk RNASeq profile differential gene expression analysis on COVID-19 patient samples to compare the difference in gene expression patterns in patients of varying infection severity and in different organ types, to analyze organ injury. The changes in gene expression between patients of progressively more severe infection may provide insight into the mechanisms of worsening conditions in patients as well as uncover possible therapeutic targets for the treatment of COVID-19 infection, which presently lacks effective treatments. In our study, we found that severe COVID-19 infection leads to the modulation of the key inflammatory genes and pathways that result in organ tissue damage in the heart, liver, lung, and kidney. The treatment of these symptoms at the organ level with the identified therapeutic targets in the inflammatory pathway axis can lead to improved prognoses for severely infected patients.

## 2. Materials and Methods

**Figure 1:**
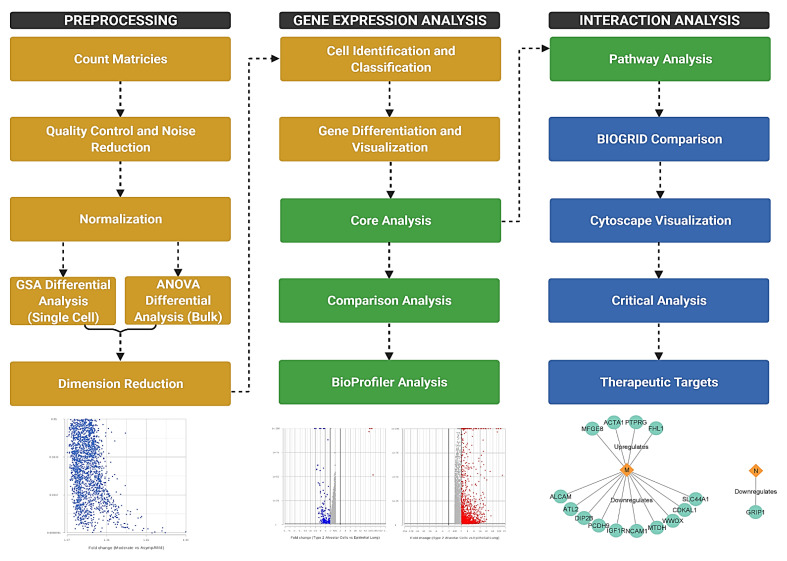
Flowchart depicting the methods of the experiment. Count matrices were imported from GEO and processed for further analysis. The three major components of the study were preprocessing, gene expression analysis, and interaction analysis. Yellow steps were performed in Partek Flow, green steps in IPA, and blue steps in other software. Generated in Biorender.

The single-cell and bulk RNA sequencing meta data sets were obtained from the National Center for Biotechnology Information’s Gene Expression Omnibus (GEO) (Table 1). Sources for samples include both autopsy samples taken from deceased patients and those from living patients with various levels of infection severity. These samples were taken from organs such as the heart, kidney, liver, and lung, as well as nasopharyngeal swabs. We used Partek Flow (Partek Inc., Chesterfield, MO, USA), a next generation sequencing software, for processing and filtering. The overall workflow of the study consisted of the pre-processing of count matrices, gene differentiation analysis, and interaction analysis (Figure 1). The significance of differentially expressed genes was determined based on fold change (−2 to 2) and *p*-value (0.05), where higher fold change indicated a more strongly down- or upregulated gene with possibly significant implications in host physiology (Supplementary Tables S1–S4).

For single-cell RNA-seq datasets GSE171668 [12] and GSE168215 [13], we performed pre-analysis filtration via Partek’s recommended settings; we discarded cells with especially high or low counts. We used and normalized the publicly available count matrices before performing GSA differential analysis. Dataset GSE171688 was comprised of samples from multiple organ types from several patients. Significance was determined by fold change of −2 to 2 and the false discovery rate was filtered to be no higher than 0.05. This allowed only the most significantly upregulated genes through the filter for further analysis. High counts may indicate undesirable doublets while especially low counts might come from damaged or fragmented cells. To reduce the dimensionality of the dataset, we performed principal component analysis (PCA) using 20 principal components. For the bronchial dataset (GSE168215), we used the graph-based clustering task to cluster similar cells together for easier visualization down the pipeline. For excessively large datasets such as the tissue atlas (GSE171668), we did not perform clustering due to limitations with the software. We visualized datasets via Partek Flow’s Uniform Manifold Approximation and Projection (UMAP) task. Cell characterization was based on gene expression for further analysis. The pipeline for bulk RNA-seq datasets was nearly identical, except we performed ANOVA differential analysis instead of GSA. For dataset GSE150316 [14], patients with high viral load were compared to those with lower viral load. The nasopharyngeal swabs from GSE162835 [15] were compared by disease severity: mild, moderate, and severe.

We next used the QIAGEN’s Ingenuity Pathway Analysis (IPA) (QIAGEN, Hilden, Germany) to determine the most significant biological pathways. The pathways were determined by analyzing processed gene lists from Partek Flow. We then generated the top down- and upregulated canonical pathways based on the statistical significance of altered gene expression. Network maps were used to identify viral-host protein interactions by comparing the host pathways to overlays of coronavirus-related pathways. The top 100 genes per organ for both down- and upregulated gene lists were compared to BIOGRID’s public SARS-CoV-2 repository of protein interactions. We used Cytoscape software (Cytoscape Team, San Diego, CA, USA) to visualize these interactions. Finally, we ran IPA’s BioProfiler task to scan multiple gene databases and generate a list of diseases, the gene’s activity and effect on disease, and links to studies pertaining to using the particular gene as a drug target. Filtering these results to relevant symptoms associated with COVID-19 and by significance in the dataset yielded a preliminary list of promising therapeutic targets. Druggable genes were determined by significance, based on fold change of the gene, and by relevance to COVID-19 infections—such as genes known to be significantly altered in acute respiratory distress syndrome or organ injury. We used the Drug-Gene Interaction Database (DGIdb, St. Louis, MO, USA) to verify and validate information about drug–gene interactions and druggable genomes.

## 3. Results and Discussion

### 3.1. Single-Cell Immune Profiling Showed Viral Effects per Organ

**Figure 2:**
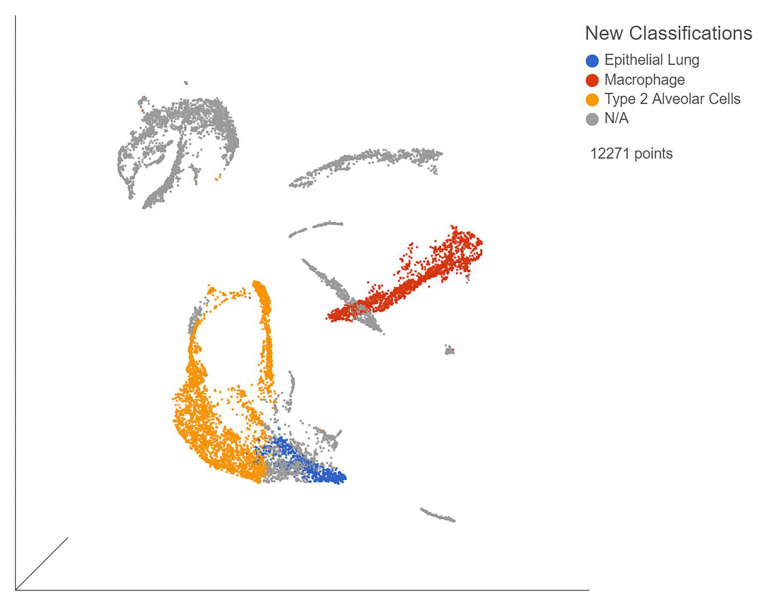
UMAP of blue points represent general lung epithelia, red points are AT2 cells, and yellow points are macrophages. Gray points represent unclassified cells. Generated in Partek Flow.

The single-cell samples provided the gene expression data for individual organ cells, allowing us to observe viral effects on each organ type. The samples (GSE168215) from bronchial brushings mostly consisted of lung epithelia (Figure 2). The biomarkers nuclear transcription factor (*NR4A1*) and mesothelin (*MSLN*) were used to identify alveolar type II cells, while general lung epithelial cells were identified by small secreted peptide (*SSP*) and formyl peptide receptor 3 (*FPR3*). These biomarkers were chosen due to being unique to their respective cell types in our analysis. When comparing severe, moderate, and mild patients, immune cell expression was determined by the use of biomarkers unique to each cell type. B cells were identified via *CD19* (Supplementary Figure S1a), macrophages by CD68 (Supplementary Figure S1b), natural killer cells by neural cell adhesion molecule 1 (*NCAM1*) (Supplementary Figure S1c), and T cells by T cell receptor-associated transmembrane adaptor 1 (*TRAT1*) (Supplementary Figure S1d). T cell expression was the lowest among all three severities. Severe patients also had decreased T cell expression when compared to moderate and mild patients. Inappropriate T cell response is associated with severe disease. Finally, we found evidence of macrophage invasion of the lung samples in dataset GSE168215.

### 3.2. Bulk RNA Sequencing Identified Differences between Severity Levels

The full names for all host gene abbreviations are provided in Supplementary Table S7. The analysis of bulk RNA-seq sample GSE150316 revealed that the top five most significantly upregulated genes for high viral load patients were: *B2M*, *N*, *IFITM1*, *HLA-C*, and *IFI6* (Supplementary Figure S2). These genes are indicative of viral-activated interferon response in the case of *IFITM1* and *IFI6*, and of viral infection as shown by presence of viral N protein. When comparing alveolar type II (AT2) cells to general lung epithelial cells, the top five most downregulated genes were *BCAM*, *KRT5*, *KRT15*, *IL-33*, and *KRT17* (Supplementary Figure S3a). BCAM is an immunoglobulin and receptor for the extracellular matrix protein laminin. *KRT5*, *KRT15*, and *KRT17* are cytokeratins involved in the structural integrity of epithelial cells. Interestingly, IL-33 is downregulated unlike most other inflammatory interleukins. Mishima et al. found that the downregulation of IL-33 leads to a delay in mucosal inflammation recovery, which could contribute to COVID-19’s deleterious effects on the lung [16]. The downregulation of structural cytokeratins and *IL-33* may contribute to the lung’s susceptibility to infection and long-haul injury. The most upregulated were *LCN2*, *CEACAM6*, *PIGR*, *KRT7*, and *BPIFB1* (Supplementary Figure S3b). LCN2 is an innate immune peptide associated with suppressing invasiveness, and BPIFB1 binds bacterial lipopolysaccharides as another element of innate immunity. PIGR is a protein that binds and transports immunoglobulins. KRT7 is a cytokeratin involved in blocking interferon-induced interphase. CEACAM6 functions by facilitating adhesion of immune cells and cytokine activation, which induces an inflammatory response, a well-documented symptom of COVID-19 infection.

**Figure 3:**
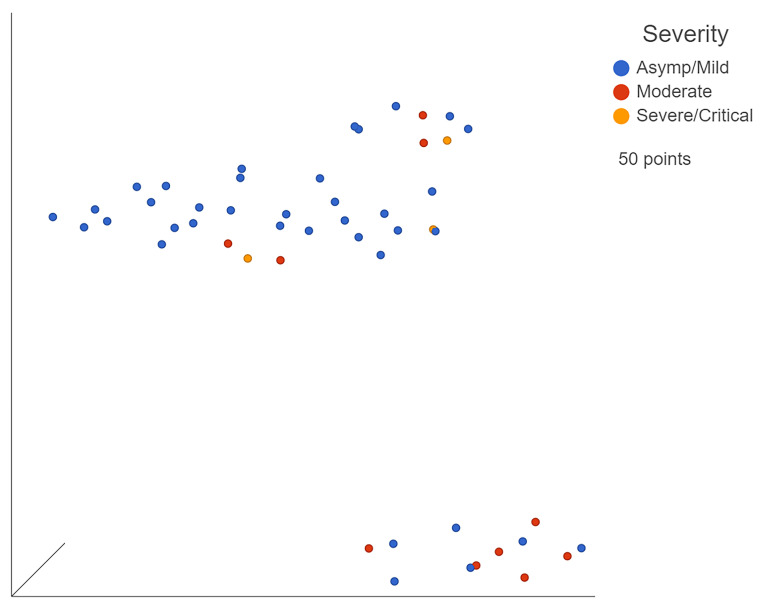
UMAP of GSE162835 classifications. Blue points represent mild, yellow moderate, and red severe patients. Generated in Partek Flow.

Bulk RNA-seq dataset GSE162835 was already sorted by severity by the original researchers (Figure 3). After processing the count matrices, the five most downregulated genes in severe patients when compared to moderate patients were *SERP1*, *DNAJC3*, *IARS2*, *CDC37*, and *PPP1R2* (Supplementary Figure S4a). The downregulation of several protective genes, specifically the interferon-induced kinase inhibitor *DNAJC3* and ER protective *SERP1*, could lead to a downstream upregulation of various inflammatory elements. This could manifest as progression of the infection to more severe states. The top five most significantly upregulated genes were *CT47A3*, *CT47A4*, *CT47A2*, *ARL10*, and *SYNPO2* (Supplementary Figure S4b). *CT47A3*, *CT47A4*, and *CT47A2* are members of the cancer/testis gene family. ARL10 is a GTP binding promoter while SYNPO2 is an alpha-actin binding enhancer. The upregulation of these promoters correlates with the increased inflammation seen in increasingly severe cases due to GTP and alpha-actin, both contributing to or resulting from inflammation, respectively.

The top five most significantly downregulated genes in severe patients when compared to mild patients were *SERP1*, *BET1L*, *DNAJC3*, *S100A16*, and *IARS2* (Supplementary Figure S5a). As with the moderate patients, protective SERP1 and kinase inhibitor DNAJC3 were downregulated in severe patients. The most upregulated genes were *CT47A3*, *CT47A4*, *CT47A2*, beta defensin (*DEFB115*), and *IGHD3-10* (Supplementary Figure S5b). Notably, beta defensin is a key part of the innate immune response against infections and *IDHD3-10* is a protein-coding gene related to immunoglobulins. These indicators of immune response were increased in severe cases when compared to milder patients.

The most highly downregulated genes in moderate patients when compared to mild were *MTRNR2L1*, *UNC79*, *ARL10*, *COL4A6*, and *TP63* (Supplementary Figure S6a). MTRNR2L1 is a protein associated with muscle disease. UNC79 encodes a subunit of a calcium channel. COL4A6 is a type of collagen IV while TP63 is a member of the p53 family involved with miRNAs in cancer. GTP binding promoter ARL10 was downregulated in this comparison, indicating that this gene is upregulated in mild and severe patients but not in moderate ones. A larger sample size would be required to determine if this finding is meaningful. The most upregulated genes were *H2BC15*, *LRRC26*, *ATP12A*, *HES4*, and *DDX3Y* (Supplementary Figure S6b). H2BC15 is a histone. LRRC26 is a protein associated with BK alpha voltage and calcium-activated potassium channels, and ATP12A is an ATPase associated with potassium absorption. Disturbances in the body’s natural potassium homeostasis can result in several different complications. Both hypokalemia and hyperkalemia can lead to arrhythmia and even fatal heart attack. Notably, the upregulated gene *HES4*—a transcription factor associated with phosphoinositide 3-kinase (PI3K)/protein kinase B (AKT) signaling—indicates an increase in PI3K signaling, a pathway that is taken advantage of by the virus for entry and development of the immune response [17].

For the bulk RNA-seq data, which measure the average expression of the samples, we found the difference in gene expression between low and high viral loads such as those in dataset GSE150316. We also used bulk data to discern the difference of expression between different levels of disease severity, as with dataset GSE162835. Thus, single-cell sequencing provided information for the cell-specific changes as a result of COVID-19 infection while the bulk data differentiated gene expression between samples of varying viral load or severity. Supplementary Table S8 shows overlapping genes in both bulk and single-cell analysis.

### 3.3. Viral Disruption of Host Translation and Related Pathways Contributes to Increased Severity

In bronchial samples from intubated SARS-CoV-2 pneumonia patients in GSE162815, the following pathways were most upregulated when considering all cell types: transforming growth factor-beta (TGF-beta) signaling, senescence, peroxisome proliferator-activated receptor alpha (PPARα)/retinoid X receptor alpha (RXRα), nuclear factor kappa B (NF-κB) signaling, and a cancer mechanism involving increased expression of oncogenes leading to cell cycle arrest and other cancer-like symptoms in bronchial cells. TGF-beta signaling and senescence pathways both lead to increased apoptosis via increased p38 mitogen-activated protein kinase (MAPK) and oncogene Ras, respectively. The PPARα/RXRα pathway is stimulated by high levels of IL-1, TGFβ, and Jun N-terminal kinase (JNK)—where JNK, specifically, could be responsible for elevated NF-κB inflammatory signaling. After classification, the AT2 cells from GSE162815 had upregulated protein ubiquitination and BAG2 signaling when compared to general lung epithelia. Both pathways involve the upregulation of proteasome 20S, a component of inflammasomes. The most strongly downregulated pathway was eIF2 signaling, a highly conserved step in host translation activation.

**Figure 4:**
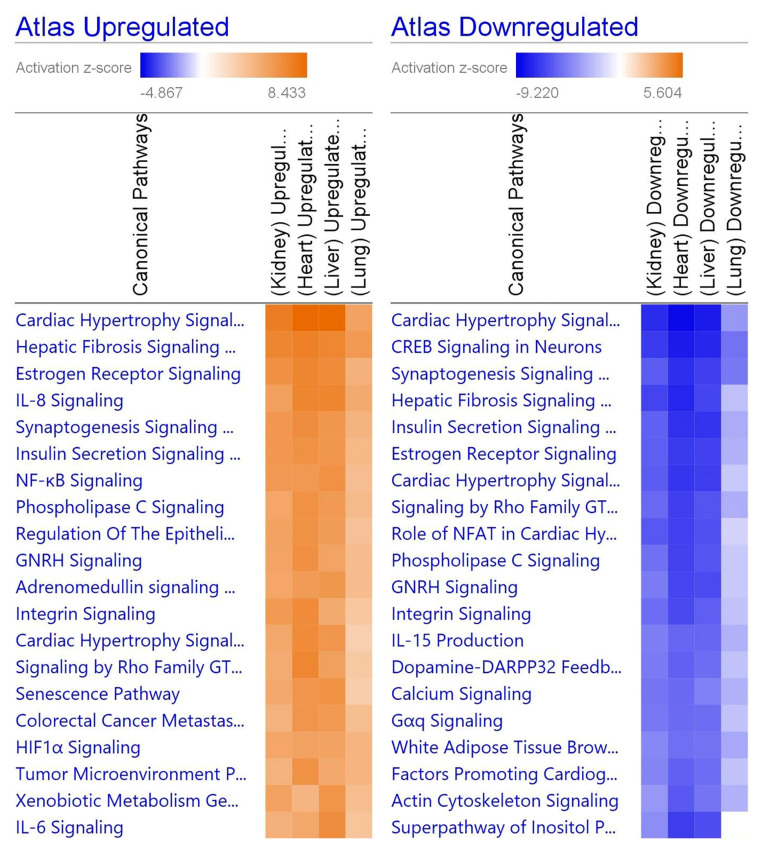
Heatmap comparing the top 20 pathways in organs. Orange indicates upregulation while blue indicates downregulation, and a stronger color indicates a stronger effect. Gray indicates that the software was unable to predict the effect. The repeated pathways are due to individually down- or upregulated steps within the pathway. Generated in IPA from gene lists.

When comparing the four organ types, various pathways unique to the particular organ were perturbed (Figure 4). For the heart, dilated cardiomyopathy was upregulated via high levels of titin (TTN), which leads to DCM. In the kidney, the complement system was downregulated via a decrease in DF enzymes, leading to less complementary system-related factors. The liver had downregulated GP6 signaling via decreased protein kinase C (PKC), leading to reduced platelet activation. Liver samples also showed upregulation in acute phase signaling, where upregulated NF-κB and STAT3 led to strongly upregulated C-reactive protein (CRP) and fibrinogen. Finally, lung tissue had decreased enzymatic function across the board, while eIF2, coronavirus pathogenesis, and Th1 signaling were all increased. Many of these pathways share common ligands, namely inflammatory cytokines and interferons.

### 3.4. Reduction of Eukaryotic Initiation Factors Leads to Increased Severity

**Figure 5:**
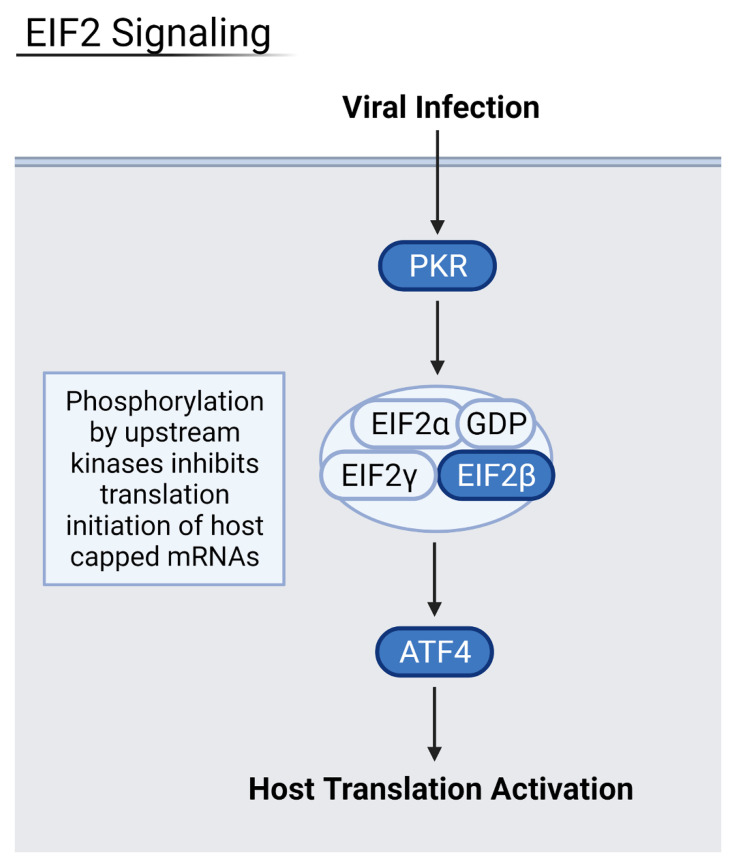
Summary of the eIF2 pathway where blue proteins are downregulated. Samples from severely infected heart, kidney, and liver shared these downregulated kinases and transcription factors, leading to an overall decrease in host translation of inflammation-inhibiting factors. Generated in Biorender.

**Figure 6:**
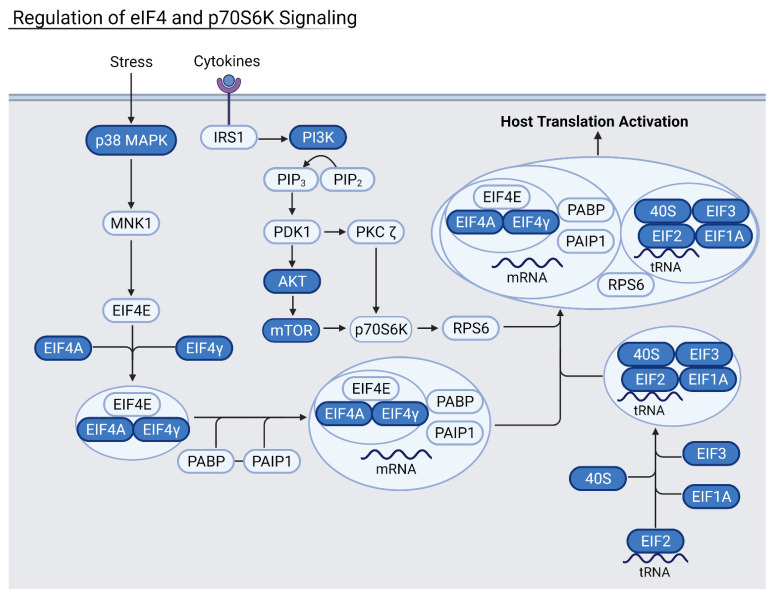
Summary of the eIF4 pathway where blue proteins are downregulated. Samples from severely infected heart, kidney, and liver shared these common downregulated factors. The eIF4 and eIF2 pathways work together to activate and enhance host translation, and their inhibition leads to increased viral translation. Generated in Biorender.

Downregulation of eukaryotic initiation factor 2 (eIF2) and eIF4 pathways was common across several datasets in moderate and severely infected patients, and in three out of the four examined organ types (Figure 5 and Figure 6). These pathways initiate the translation of capped host mRNAs and are a vital part of the immune response to viral infection. The eIF2 pathway also contributes to inhibition of the NF-κB pathways and pro-inflammatory factors [18]. NF-κB proteins are transcription factors that control inflammatory responses and lead to a detrimental inflammatory response if left uninhibited. Patients with increasingly severe infection saw more downregulated eIF2 and eIF4 signaling, hallmarked by decreased levels of eIF peptides and necessary host transcription factors such as N-MYC. Critically ill patients, especially, saw downregulation of eIF3 and eIF4 proteins as well as important kinases such as PI3K and protein kinase R (PKR). Thus, the downregulation of these factors may lead to increased severity of disease.

Heart samples showed downregulation of these vital pathways via a decrease in essential eIF-related kinase PKR as well as the initiation factors themselves. Kidney samples from severe patients had downregulation of eIF2 and eIF4 pathways via the downregulation of protein kinase R-like ER kinase (PERK), a necessary upstream step for eukaryotic activating transcription factors, namely activating transcription factor 5 (ATF5) and ATF3. Liver samples also had downregulation of eIF2 and eIF4 pathways, which led to an increase in pro-inflammatory NF-κB. NADPH oxidase 4 (NOX4) oxidase was especially downregulated, leading to a decrease in the protective response against inflammation as well as a decrease in anti-apoptotic regulator BCL2. In conjunction with eukaryotic initiation factor downregulation, the mTOR signaling pathway was also downregulated in severe and deceased patients. As an upstream step of host translation, alterations in this pathway have downstream effects that decrease translation. In heart, kidney, and liver samples from severe patients, decreased PI3K, eIF peptides, and mTORC1 and mTORC2 complexes were observed. The eIF4 pathway relies on mTORC1 to activate the eukaryotic translation initiation factor complex. As a result, the heart and kidney seem especially susceptible to COVID-19 infection via the viral suppression of host translation.

Interestingly, eIF2 signaling was highly upregulated in severe lung samples. This could be explained by the interaction of virus proteins with eIF2α, a regulatory node of the eIF2, as explored by Liu et al., who found that RNA viruses can phosphorylate eIF2α to provide a favorable environment for their own translation [19]. SARS-CoV-2 protein SARS3A was shown to interact with eIF2α which, in turn, stimulates activating transcription factor 4 (ATF4). ATF4 is a pro-apoptotic host transcription factor that can assist in viral pathogenesis. PKR kinase, a regulatory early step in the eIF2 pathway, is thought to be highly stimulated in the early stages of viral infection to increase pathogenesis and, as the infection progresses to a more severe state, PKR is downregulated to curtail host translation. The lung’s eIF2 signaling is used by the virus for its own proliferation and, thus, is not downregulated as seen in other organs.

### 3.5. Inflammatory Interleukin Levels Increase with Severity

**Figure 7:**
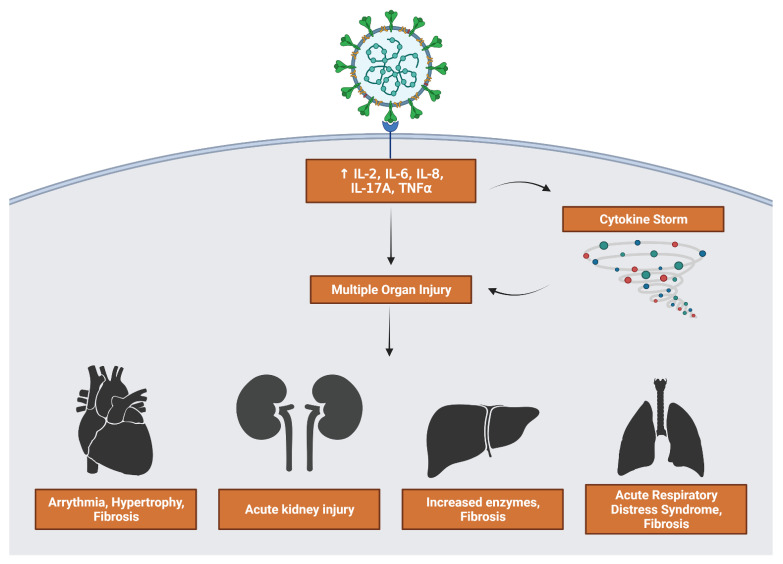
Consequences of viral stimulation of inflammatory factors. Upregulation of cytokines feeds directly into multiple organ injury as well as activating a cytokine storm that further contributes to injury. Generated in Biorender.

A hallmark of COVID-19 infection, especially severe infection, is a loss of control over immune response leading to increased inflammation. Dangerous symptoms such as acute respiratory distress syndrome and multiple organ failure can occur as a result (Figure 7). The upregulation of interleukin signaling in severe and moderate patients can lead to a positive feedback loop where more interleukins and cytokines are released. Severe patients had a marked increase in IL-2 and IL-17 expression. Heart, kidney, liver, and lung organ samples all showed upregulated IL-6 and IL-8 signaling and their downstream effects.

IL-2 is a cytokine that is a part of the immune response to infection. It mostly activates leukocytes via IL-2 receptors on their cell surface, stimulating inflammation to combat the infection. It is transduced via the PI3K/Akt and JAK-STAT pathways, all of which were upregulated in severe patient samples. Furthermore, the upregulation of inflammatory transcription factors such as NFAT and NF-κB leads to increased IL-2 levels, leading to a positive feedback loop where pro-inflammatory cytokines continue to upregulate each other. To further support IL-2’s role in COVID-19 severity, the use of recombinant IL-2 (rIL-2) by Zhu et al. stimulated lymphocyte recovery in severe patients. The recombinant interleukin, which has been used in cancer patients to reactivate their immune systems, was found to enhance the antiviral effect in mice in prior studies [20]. IL-6 and CRP levels were also decreased after rIL-2 treatment compared to untreated patients.

**Figure 8:**
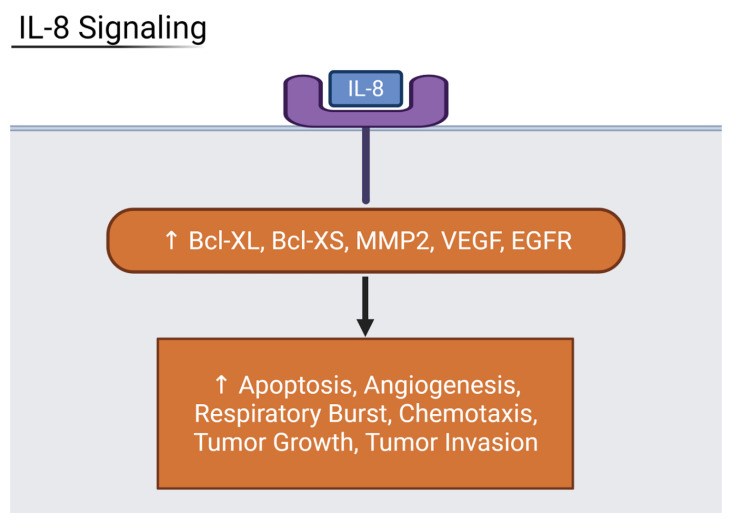
Diagram showing IL-8 downstream effects on organs. Inflammatory IL-8 upregulates host factors that contribute to cell death, angiogenesis, respiratory burst, and more. Generated in Biorender.

Among moderate and severe patients, IL-17 signaling was increased with marked upregulation in beta defensin peptides and pro-inflammatory cytokines when compared to mild and asymptomatic patients. Shibabaw et al. found that the cytokine release syndrome relied on IL-17A upregulation, a component of the cytokine storm and immunopathy of COVID-19 [21]. IL-6 binding activates the JAK/STAT3 pathway, and results in the production of inflammatory cytokines including IL-17A. IL-6 is a cytokine with some anti-inflammatory effects in its ability to inhibit TNFα. However, in the case of severe COVID-19 patients, it often serves only to increase the levels of CRP via the JAK-STAT pathway. All four organ types and the liver, especially, have highly increased levels of CRP, a non-specific acute phase protein that correlates to disease severity in COVID-19 [22]. This makes IL-6 an inflammatory marker for severe COVID-19. The final interleukin that showed the highest upregulation was IL-8. The heart and liver, specifically, are strongly affected by increased IL-8 expression. The liver showed signs of upregulated NF-kB activity, leading to increased transcription of angiogenic peptides ICAM-1 and VCAM-1. The heart had increased expression of apoptotic factors Bcl-XL and Bcl-XS, and angiogenic factors MMP2 and VEGF (Figure 8). As a result, IL-8 is also considered a biomarker for severe disease prognosis in COVID-19 patients [23].

### 3.6. Suppression of Host Translation Leads to Increased Inflammation

**Figure 9:**
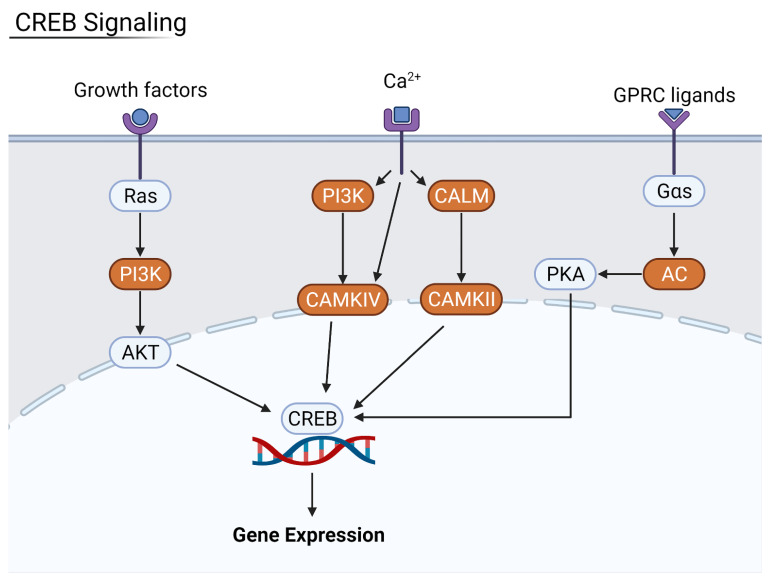
Summary of the CREB signaling pathway. Key kinases such as PI3K were upregulated in more severe patients, leading to an increase in CREB-directed gene expression. Generated in Biorender.

Nuclear factor kappa-light-chain-enhancer of activated B cells, often shortened to NF-κB, is a transcription factor involved with cytokine production. It is strictly regulated by pathways such as eIF2 translation of its inhibitory factors, and loss of this inhibition can lead to autoimmune and inflammatory disease. This pathway was upregulated in severe patients and in all four organ types. This is due to increased levels of pro-inflammatory IL-1 and tumor necrotic factor α (TNFα); Ras GTPase activity was upregulated, leading to the p65/RelA–NF-κB complex inducing the transcription of more inflammatory peptides. As a consequence of decreased host translation, regulatory factors that ordinarily suppress immune response are lost, and the immune system is activated in an attempt to combat the infection and restore homeostasis. One such pathway that becomes upregulated is the cAMP response element-binding (CREB) signaling pathway. CREB is a transcription factor involved in cellular response to stimuli that expresses certain genes in response to activation by inflammatory cytokines such as *IL-2* and *IL-6* [24]. CREB signaling was found to be highly upregulated in severe patients when compared to moderate and mild patients. As a result of the upregulation, there was an increase in pro-fibrotic factors alpha-actin (ACTA2) and collagen type 1 in all four organ types (Figure 9).

### 3.7. Inflammation Results in Hypertrophy and Fibrosis

**Figure 10:**
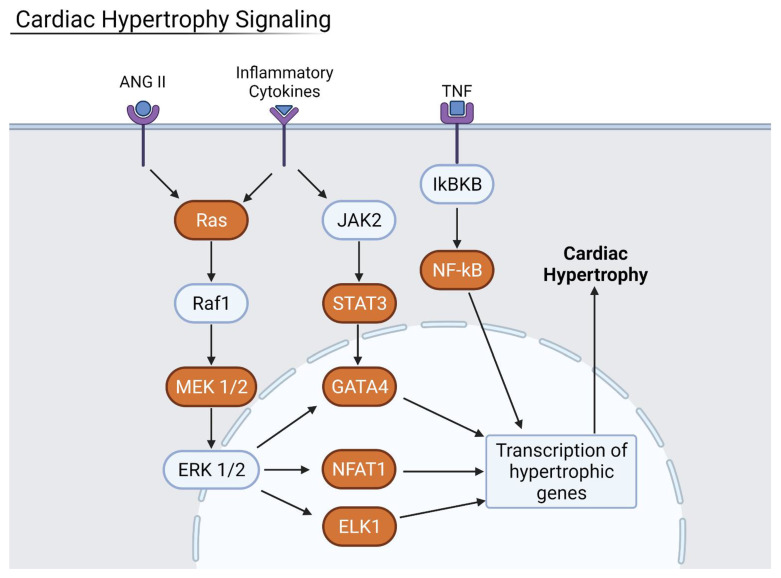
Cardiac Hypertrophy pathway where orange proteins are upregulated. ANGII, inflammatory cytokines, and TNF—all increased during COVID-19 infection—upregulated the cardiac hypertrophy signaling pathway. Generated in Biorender.

In all four organ types, hypertrophic and fibrotic factors were found to be highly upregulated. Signaling proteins of the small GTPase family such as Ras and RHOA are activated by the binding of cytokines, ultimately leading to the downstream effects of increased hypertrophy and fibrosis (Figure 10). Cardiac hypertrophy is the thickening or enlargement of the heart that is often associated with heart failure and death. It can be exacerbated by increased NF-κB expression of inflammatory cytokines in the heart. Heart samples from severe COVID-19 patients had increased levels of heat shock protein 27 (HSP27), a known hypertrophic factor and oxidative stressor.

**Figure 11:**
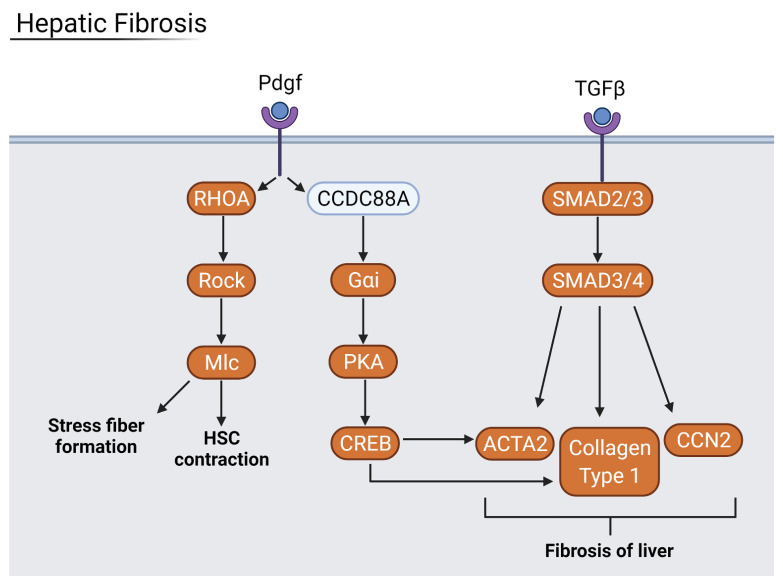
Hepatic Fibrosis pathway where orange proteins are upregulated. COVID-19 infection stimulates the fibrotic pathway via the upregulated genes. Generated in Biorender.

Hepatic fibrosis is a wound healing response to liver injuries such as autoimmunity, alcohol abuse, and viral infection. It involves the transition of hepatic stellate cells (HSCs) from an inactive state to a proliferative and migratory state in order to facilitate fibrogenesis. The formation of excessive connective scar tissue in the liver can result in cirrhosis, leading to further liver complications. The liver samples from severe COVID-19 patients showed signs of fibrosis via RHOA GTPase and CREB-directed gene expression (Figure 11). Fibrotic factors such as *ICAM-1*, *VCAM-1*, collagen type 1, and *SERPINE1* were strongly upregulated in liver and lung samples, indicating lung fibrosis as a result of viral infection as well. Lung fibrosis is a well-known precursor to acute respiratory distress syndrome (ARDS), an often-fatal lung condition where fluids leak into the lungs. The lung fibrosis and damage associated with severe COVID-19 infection can lead to the development of ARDS [3,25]. Therefore, the prevention of fibrosis is vital to improve the prognoses of severe COVID-19 patients.

### 3.8. Interaction Mapping Shows Viral Proteins Disrupt Host Translation

The top 100 down- and upregulated genes per each organ were narrowed down to 10 of the most prolific genes across all organ types. All four organs saw downregulation of *APOB*, *NCAM1*, *PCDH9*, and *ZNF536*. *APOB* was acted upon by a viral protein NSP6. *NCAM 1* interacted with viral E, M, ORF7a, and ORF7b proteins. *PCDH9* interacted with M, NSP4, NSP6, ORF3a, ORF3b, ORF6, ORF7a, ORF7b and S proteins. *ZNF536* interacted with NSP7 and NSP16. For heart, liver, and lung, the most common genes were glutamate receptor-related *GRIP1*, *UGT8*, and *FRMD5*. *GRIP1* was interacted with by viral N exclusively. UDP-glycosyltransferase *UGT8* was acted upon by E, NSP4, NSP6, ORF3a, ORF7a, ORF7b, and ORF8. *FRMD5* interacted only with ORF7b. In the heart, kidney, and liver, the host genes *KNOP1* and *MARCHF1* were downregulated. They are known to interact with viral proteins NSP2 and ORF14, and with NSP6, NSP7, and ORF9b, respectively. KNOP1 is a nucleolar protein that interacts with zinc finger proteins while MARCHF1 is a ubiquitin ligase known to downregulate surface expression of MHC class II molecules. Finally, among heart and liver samples, *ERBB4*, a tyrosine protein kinase involved with PKA activation, was downregulated. *ERBB4* is known to interact with viral protein ORF7b. In all four organs, genes that were commonly downregulated by viral proteins pertained to decreasing immune protection and increasing the harmful inflammatory effect.

### 3.9. Commonly Altered Host Genes Correlate with Disease Symptoms

Via single-cell RNAseq comparison of all four organs, *APOB*, *PCDH9*, and *NCAM1* were found to be the most commonly downregulated and had the most possible interactions with viral proteins. *APOB* codes for apolipoprotein-B, one of many apolipoproteins known to be decreased in the plasma of COVID-19 patients [26]. *PCDH9* protocadherin plays an essential role in epithelial cell–cell adhesion and the integrity of endothelial barrier function. Fogeron et al. hypothesize that the disruption of cadherin proteins by viral protein ORF7b could contribute to several symptoms of COVID-19 infection including multiorgan failure [27]. *NCAM1* is involved in the expansion of immune cells such as B cells, T cells, and natural killer cells. Its interaction with fibroblast growth factor receptors can trigger signaling cascades related to the PI3K/AKT and MAPK signaling pathways. The PI3K/AKT pathway, especially, plays a key role in COVID-19 infection, where it is involved with viral entry and NF-κB activation, which eventually leads to increased inflammatory cytokine production and fibrosis [17]. The MAPK pathway also mediates the release of inflammatory cytokines and is known to directly interact with a SARS-CoV-2 viral protein in such a way that the virus hijacks p38 MAPK to promote its own replication [28].

In heart and liver samples, the *ALCAM/CD166* gene was downregulated. *ALCAM* functions by binding to the T cell differentiation antigen CD6. This pathway leads to the increased production of inflammatory cytokines. Filgueira et al. found that the use of CD6 antibody Itolizumab led to decreased cytokine release in severe and critically ill COVID-19 patients [29]. In kidney samples, MHC class I-associated *B2M* was downregulated. Low B2M correlates to renal damage and cardiovascular disease. Conversely, in a study by Conca et al., circulating serum B2M levels were found to be increased in COVID-19 patients, where increased levels were associated with increased disease severity [30]. The genes most upregulated in heart samples are often related to myopathy, such as *ACTA1* and *SGCD*. Inflammatory myopathy in respiratory and cardiac systems, especially, are prevalent in severe COVID-19 cases. The *FHL1* gene, which is related to the JAK-STAT pathway, is also upregulated in heart samples. The phosphorylation of JAK leads to the activation of STATs, which translocate to the nucleus of the cell and translate various inflammatory mediators [31]. This increase in inflammation corresponds to the worsening of symptoms in more severe infections. Finally, in the lung specifically, *PDE3B*—a dual function second messenger for cAMP and cGMP—is upregulated. Part of its function is to regulate the binding of cAMP to RAPGEF3, which will then complex with PIK3R6 to induce angiogenesis. This formation of new blood vessels can contribute to the development of ARDS.

SARS-CoV-2 viral proteins exhibit extensive interaction with host proteins. Viral proteins E, M, NSP4, and ORF7b were the most interactive, often interacting with host proteins pertaining to immunity and host translation in all organ types. The top three genes with the most viral-host protein interactions were *APOB*, *PCDH9*, and *NCAM1*—all proteins that correspond to COVID-19 symptoms such as decreased apolipoproteins, decreased endothelial barrier function, and decreased expansion of immune cells (Table 2). Finally, viral proteins were also found to interact with host genes pertaining to cardiomyopathy and angiogenesis.

### 3.10. Individual Organs Have Unique Viral Interactions

Among the downregulated heart gene list (Supplementary Figure S7), the most interactable genes are *CDKAL1*, *WWOX*, *ALCAM*, *MTDH*, and *PCDH9*. These genes mediate anticodon–codon interaction during translation as well as tRNA processing and interacting with viral proteins E, M, NSP2, NSP3, NSP4, NSP6, ORF3a, ORF6, ORF7a, ORF7b, ORF8, ORF14, and S. *WWOX* is a short chain oxidoreductase with transcriptional functionality that can be altered by viral proteins E, M, NSP4, NSP6, ORF3a, ORF3b, ORF6, ORF7a, ORF7b, and S. Immunoglobulin receptor *ALCAM* was acted upon by viral proteins M, NSP4, ORF3b, ORF7a, ORF7b, ORF9c, ORF14, and S. Interestingly, NF-κB activator *MTDH*, which has interactions with viral proteins E, M, NSP2, NSP4, NSP6, ORF7a, ORF7b, ORF8, and S, was downregulated. Conversely, the most commonly interacted-with upregulated gene was *PTPRG*, a protein tyrosine phosphatase involved with differentiation and oncogenic transformation. It was affected by viral proteins M, NSP4, ORF3a, ORF3b, ORF7b, and S. *MFGE8* interacted with viral proteins M, NSP4, NSP6, ORF3a, and ORF8, and is known to promote phagocytosis of apoptotic cells. *FHL1* is related to JAK-STAT signaling and is known to interact with viral proteins E, M, and ORF7a. The last two most commonly interacted-with upregulated genes were *ACTA1* and *SGCD*, which have to do with fiber defects and cardiomyopathy, respectively. The most interacted-with viral proteins were M, NSP4, ORF7b, and ORF8. Overall, some of the most downregulated genes in heart samples include those pertaining to immune protection, while the most upregulated have roles in heart damage via NF-κB disruption, fiber promotion, and cardiomyopathy.

In kidney samples, *PCDH9* was one of the most commonly interacted-with downregulated genes, as with the other organs (Supplementary Figure S8). Transmembrane protein *CRIM1* was the next most interacted-with downregulated gene, interacting with viral proteins M, NSP4, NSP6, ORF3a, ORF3b, ORF7a, ORF7b, and ORF8. MHC class I heavy chain protein *B2M* was also suppressed in kidney samples. Its known host-viral protein interactions include those with viral proteins M, ORF3a, ORF7a, ORF7b, and ORF8. Heat shock protein *HSPA1A* is related to ischemia and other diseases, and it has interactions with viral proteins E, M, N, NSP4, NSP6, and ORF9b. For the final top downregulated gene, netrin receptor *UNC5C* contains immunoglobulin-like domains that interact with viral proteins E, M, NSP4, ORF3a, and ORF3b. For the most upregulated genes in kidney samples, *WDR72* was among the most highly interacted with genes—it was found to network with viral proteins E, M, NSP4, NSP6, NSP7, NSP8, NSP9, ORF3a, ORF6, ORF7a, ORF7b, ORF8, ORF9b, ORF14, and S. The *SYNE2* nuclear membrane protein is involved in cell cycle regulation and is acted upon by viral proteins E, M, NSP4, NSP6, ORF6, ORF7a, ORF7b, ORF8, and S. Translational control is altered via upregulation of *CA12*, a zinc metalloenzyme that interacts with viral proteins M, NSP4, NSP6, NSP7, ORF3a, ORF3b, ORF6, ORF7a, ORF7b, and ORF8. Oxidoreductase *OXR1* was also upregulated and is known to interact with viral proteins N, NSP2, NSP4, NSP6, NSP15, ORF7a, and ORF7b. Finally, ECM protein *FRAS1* was also upregulated in kidney samples and can interact with viral proteins NSP4, NSP6, ORF3a, ORF7a, and ORF7b. As with heart samples, kidney samples saw downregulation of protective genes while those that could be hijacked for viral use were upregulated. Increasing translation and cell cycle controllers could create a favorable environment for viral proliferation in kidney tissue.

Among the liver samples, the top five most downregulated host genes were commonly associated with host transcription and translation (Supplementary Figure S9). Similar to the heart, *CDKAL1*, *WWOX*, *ALCAM*, and *MDTH* were the most downregulated. As with the other organ samples, *PCDH9* was also downregulated in liver samples. The most upregulated gene in liver samples was *SLC39A14*, a metal transporter involved with ferroptosis—a form of apoptosis associated with COVID-19 infection [32]. *SLC39A14* interacted with viral genes E, M, NSP4, NSP5, NSP6, NSP13, ORF3a, ORF3b, ORF6, ORF7a, ORF7b, and S. Endoplasmic reticulum membrane protein *POR* interacts with viral proteins E, M, NSP2, NSP4, NSP6, ORF3a, ORF6, ORF7a, ORF7b, ORF8, ORF14, and S. Membrane-bound proteins *SCARB1* and *SL7A2* were also upregulated in the liver samples and saw marked interaction with viral proteins E, M, NSP4, NSP6, NSP7, ORF3a, ORF3b, ORF7a, ORF7b, ORF8, ORF14, and S. Finally, host *SMOC1* is a calcium ion binder known to interact with viral proteins E, M, ORF3b, ORF7b, and ORF8. One of the most commonly interacted-with genes in liver samples was *SLC39A14*, a gene involved with ferroptosis, which could lead to worsening prognoses in COVID-19 infection.

In the lung, the most commonly downregulated genes were related to receptor binding (Supplementary Figure S10), such as *GRID1* and *MAP7*, or interferon signaling, in the case of *NCAM1*. SARS-CoV-2 proteins M, NSP4, and ORF7b interact with *GRID1*, while N and ORF8 interact with *MAP7*. *UGT8*, a UDP-glycosyltransferase involved in sphingolipid metabolism, was also downregulated. It is known to interact with viral proteins E, NSP4, NSP6, ORF3a, ORF7a, ORF7b, and ORF8. Finally, the commonly downregulated *PCDH9* was the most downregulated protein in lung samples. For upregulated genes, HLA class I heavy chain paralogues *HLA-A* and *HLA-C* were commonly interacted with by viral proteins E, M, NSP4, NSP6, ORF3a, ORF3b, ORF7a, ORF7b, ORF8, and S. *RNF149* ligase was also upregulated in lung samples and has interactions with E, M, NSP3, NSP4, NSP6, ORF7a, ORF7b, ORF8, and S. Autophagy-related transmembrane protein *VMP1* can be affected by viral proteins E, M, NSP3, NSP4, NSP6, ORF7a, ORF7b, ORF8, and S. *PDE3B* is a host gene related to cAMP and cGMP signaling, and it can be stimulated by viral proteins E, M, NSP4, NSP6, ORF7a, ORF7, and S. For lung samples, receptor-binding and interferon signaling genes were suppressed by viral proteins. HLA class genes, in particular, play a role in immunity and could serve as potential therapeutic targets.

### 3.11. Potential Therapeutic Targets

Using IPA’s BioProfiler, we generated a list of genes and their effects on diseases and biological functions, as well as drug target evidence. Cross-referencing this list with the most significantly altered genes from the samples, we created a table of promising drug targets (Table 3). The complete list of all potential targets includes those for the treatment of COVID-19 infection and organ damage such as hypertrophy and fibrosis (Supplementary Tables S5 and S6). Selection criteria for potential therapeutic targets included druggability, fold change, and known effects on diseases or symptoms. Targets involved in the treatment of other coronaviruses were also given importance. The targets were sorted by evidence of targetability including unverified targets that have yet to be tested for any condition, those that are verified for use in other diseases, and those confirmed for use in COVID-19 that are currently undergoing evaluation. Significantly enhanced unverified targets include *ANPEP, CCL2*, *HBB*, *IFI27*, *HLA-A*, *HLA-B*, and *HLA-C*. The HLA series of genes have been proposed as therapeutic targets for many diseases due to their variability and involvement in immunity; however, they have yet to be tested beyond in silico investigation. Interferon alpha inducible protein 27 (IFI27) was also selected as a noteworthy target due to its upregulation in COVID-19 infection and its relation to interferon- and immune-related pathways. Targets with known use in other diseases include *IL-17A*, *IL4R*, *ALPI*, and *MAPK8*. Finally, enhanced targets already undergoing evaluation for COVID-19 treatment include *SERPINC1*, *CCL7*, *AGTR1*, *TUBG2*, *PPARG*, *IL-6*, and *GABRA4*. Of these targets, interleukins 6 and 17A are recommended for further testing due to their ability to trigger many deleterious downstream effects in severe COVID-19 infection. *IL-6* and *IL-17A* were among the most upregulated genes in our study. Interleukin receptor *IL4R* is commonly upregulated in pulmonary diseases and treatments have proven successful in other diseases. *ALPI* is another component of immune defense that can be targeted to treat disease in general. We identified many targets for organ-specific treatments as well. Genes such as *MAPK8*, *AGTR2*, and *SERPINC1* could serve as liver-related therapeutics while *PPARG* could be used in kidney treatment as well as SARS. Heart-related genes include *NR3C2*, *JUN*, and *CIB1*. We also verified existing potential COVID-19 drug targets, such as *SERPINC1* and the inflammatory cytokine *CCL7*, that are already undergoing testing. Due to selecting via fold change and existing research, it is possible that entirely novel gene variations were overlooked. Though the list requires further analysis and may need expansion in the future, we propose these potential host genes as therapeutic targets for COVID-19 infection.

## 4. Conclusions

In this study, to understand the mechanisms of multiple organ failure and infection severity of COVID-19 patients, we assessed single-cell and bulk RNA-seq samples to find genes and pathways altered by the virus. The results of this study validated the current literature, suggesting that a progression of disease severity leads to an increase in injurious symptoms for COVID-19 patients. The upregulation of inflammatory cytokines such as *IL-2*, *IL-6*, *IL-8*, and *IL-17A* correlated with higher severity of infection and more extreme symptoms such as organ injury. Common methods of viral interference include inhibition of host translational pathways, downregulation of host antiviral measures, and upregulation of interferon-induced peptides via inflammatory cytokines. For the four organ types explored in this study, the most commonly upregulated pathways included interleukin signaling, NF-κB transcription-mediated transcription, and injurious pathways such as cardiac hypertrophy and hepatic and pulmonary fibrogenesis. Mild, moderate, and severe patients can have distinct symptoms from each other. While an increase in severity correlated with an increase in critical symptoms such as organ damage and acute respiratory distress syndrome, unique symptoms such as loss of taste can occur in moderate but not mild or severe patients, suggesting that susceptibility to some symptoms is based on individual host gene variation. Our RNA-seq analysis showed the upregulation of inflammatory genes and downregulation of protective genes in infected hosts. Pathway analyses of these gene lists indicated that viral proteins disrupted host translation and related pathways. The suppression of host translation led to increased inflammation and the upregulation of inflammatory interleukins. As a result of this inflammation, organs displayed signs of hypertrophy and fibrosis. Interaction mapping demonstrated that viral proteins disrupt host processes and which specific proteins target host translation. Individual organ types also have unique host–virus protein interactions. Commonly altered host genes, such as *PCDH9* and *NCAM*, related to immune protection were downregulated in all tested organ types and correlated with COVID-19 symptoms such as organ failure and reduced immune cell expansion. Finally, we identified several potential therapeutic targets based on the significance of differential expression of a given gene and its druggability. We found various promising targets from a range of unverified genes, genes used in the treatment of other diseases, and verified genes already being tested for COVID-19 treatment.

Our study investigated down- and upregulated genes in COVID-19 patients and their effect on host pathways leading to organ damage. We mapped which coronaviral proteins interact with the identified host proteins. Finally, we identified host genes for potential therapeutic targets. Additional research could verify the potential host therapeutic targets. The study could be further expanded to include more organ types such as the nervous system or the gastrointestinal tract, which has ACE2 receptors on its surfaces.

## Figures and Tables

**Table 1:** RNA-seq datasets. Each publicly available dataset’s accession number, RNA-seq type, and information about each study.

GEO Accession	Study Design	Sample Source	Sample Size	Number of COVID Patients	Number of Healthy Control	Type
GSE171668 [12]	COVID-19 autopsy biobank of 420 autopsy specimens, spanning 11 organs and 17 donors.	Various organs (lung, kidney, liver, heart)	118	17	0	Single-cell RNA-seq
GSE168215 [13]	Bronchial brushings from 4 patients with COVID-19 were obtained at Northwestern Memorial ICU.	Lung	9	4	0	Single-cell RNA-seq
GSE150316 [14]	Autopsy samples from patients deceased due to SARS-CoV-2 infection were collected for total RNA-seq analysis to assess viral load and immune response.	Lung, liver, heart, jejunum, bowel, kidney, placenta	88	5	5 samples (patient # not indicated)	Bulk RNA-seq
GSE162835 [15]	Shotgun transcriptome sequencing of human RNA obtained from nasopharyngeal swabs of patients with COVID-19.	Nasopharyngeal swabs	50	50	0	Bulk RNA-seq

**Table 2:** Genes and Their Role in Severity. A list of the top genes and their function in disease severity.

Factors of Severity	Genes and Function
Decreased Immune Response	*HLA-A, -B, -C*—immune regulatory genes *NCAM1*—decreased immune cell expansion and interferon signaling
Factors that Correlate with Severity	*APOB*—decreased apolipoproteins COVID-19 patient plasma *B2M*—decreased organ expression contributes to renal and cardiovascular damage
Fibrosis	*ACTA1*—pro-fibrotic factor
Increased Hypercytokinemia	*ALCAM*—increased cytokine production *FHL1*—increased cytokine translation
Increased Viral Transcription and Translation	*CA12*—translational control *SYNE2*—contributes to cell cycle regulation
Multi-Organ Failure	*PCDH9*—cadherin disruption decreases endothelial barrier integrity
Organ Specific Damage	*HSPA1A*—ischemia and kidney damage *PDE3B*—induces angiogenesis, contributes to ARDS *SGCD*—related to cardiomyopathy *SLC39A14*—related to ferroptosis in liver

**Table 3:** Potential Therapeutic Targets. Possible COVID-19 therapeutic targets based on targetability in similar diseases and prevalence in COVID-19 infection.

Gene	Basic Functionality	Drug Target Evidence
*ANPEP*	Peptidase with increased activity in coronaviral infections	Unverified
*CCL2*	Inflammatory cytokine upregulated in many viral infections	Unverified
*HBB*	Hemoglobin beta subunit that regulates inflammatory cytokines	Unverified
*IFI27*	Interferon-induced protein upregulated in severe COVID-19	Unverified
*HLA-A*	MHC Class I peptide upregulated in COVID-19 and liver injury	Unverified
*HLA-B*	MHC Class I peptide upregulated in COVID-19 and liver injury	Unverified
*HLA-C*	MHC Class I peptide upregulated in COVID-19 and liver injury	Unverified
*IL-17A*	Cytokine that is upregulated in severely infected patients	Use in other diseases
*IL4R*	Cytokine receptor that is upregulated in pulmonary disease	Use in other diseases
*ALPI*	Phosphatase that is upregulated in respiratory disease	Use in other diseases
*MAPK8*	Kinase involved in apoptosis in SARS infections	Use in other diseases
*SERPINC1*	Upregulated activity in cirrhosis of liver and COVID-19	Validated COVID-19 Use
*CCL7*	Inflammatory cytokine	Validated COVID-19 Use
*AGTR1*	Increased activity appears in cirrhosis and SARS infections	Validated COVID-19 Use
*TUBG2*	Structural molecule upregulated in viral infections	Validated COVID-19 Use
*PPARG*	Receptor upregulated in kidney injury and SARS infections	Validated COVID-19 Use
*IL-6*	Pro-fibrotic factor and inflammatory cytokine	Validated COVID-19 Use
*GABRA4*	GABA receptor with increased expression in SARS infections	Validated COVID-19 Use

## Supplementary Materials

The following are available online at https://www.mdpi.com/article/10.3390/v13122418/s1, Figure S1: GSE162835 Immune Cell Plots, Figure S2: GSE150316 Volcano Plot of High versus Low Viral Load, Figure S3: GSE168215 Volcano Plots, Figure S4: GSE162835 Fold Change—Severe v Moderate, Figure S5: GSE162835 Fold Change—Severe v Mild, Figure S6: GSE162835 Fold Change—Moderate v Mild, Figure S7: Host-Viral Interactions—Heart, Figure S8: Host-Viral Interactions—Kidney, Figure S9: Host-Viral Interactions—Liver, Figure S10: Host-Viral Interactions—Lung, Table S1: GSE171668, Table S2: GSE168215, Table S3: GSE162835, Table S4: GSE150316, Table S5: BioProfiler GSE168215, Table S6: BioProfiler GSE171668, Table S7: Gene Names, Table S8: Gene Table Comparison Bulk and SC.
