# Supplementary material for: Single-Cell and Bulk RNASeq Profiling of COVID-19 Patients Reveal Immune and Inflammatory Mechanisms of Infection-Induced Organ Damage
Source: Viruses. 2021 Dec 2;13(12):2418. doi: 10.3390/v13122418 (PMC8706409; doi:10.3390/v13122418)
Supplement: Supplementary file 1 [file viruses-13-02418-s001.zip › Supplementary Figures.pdf]

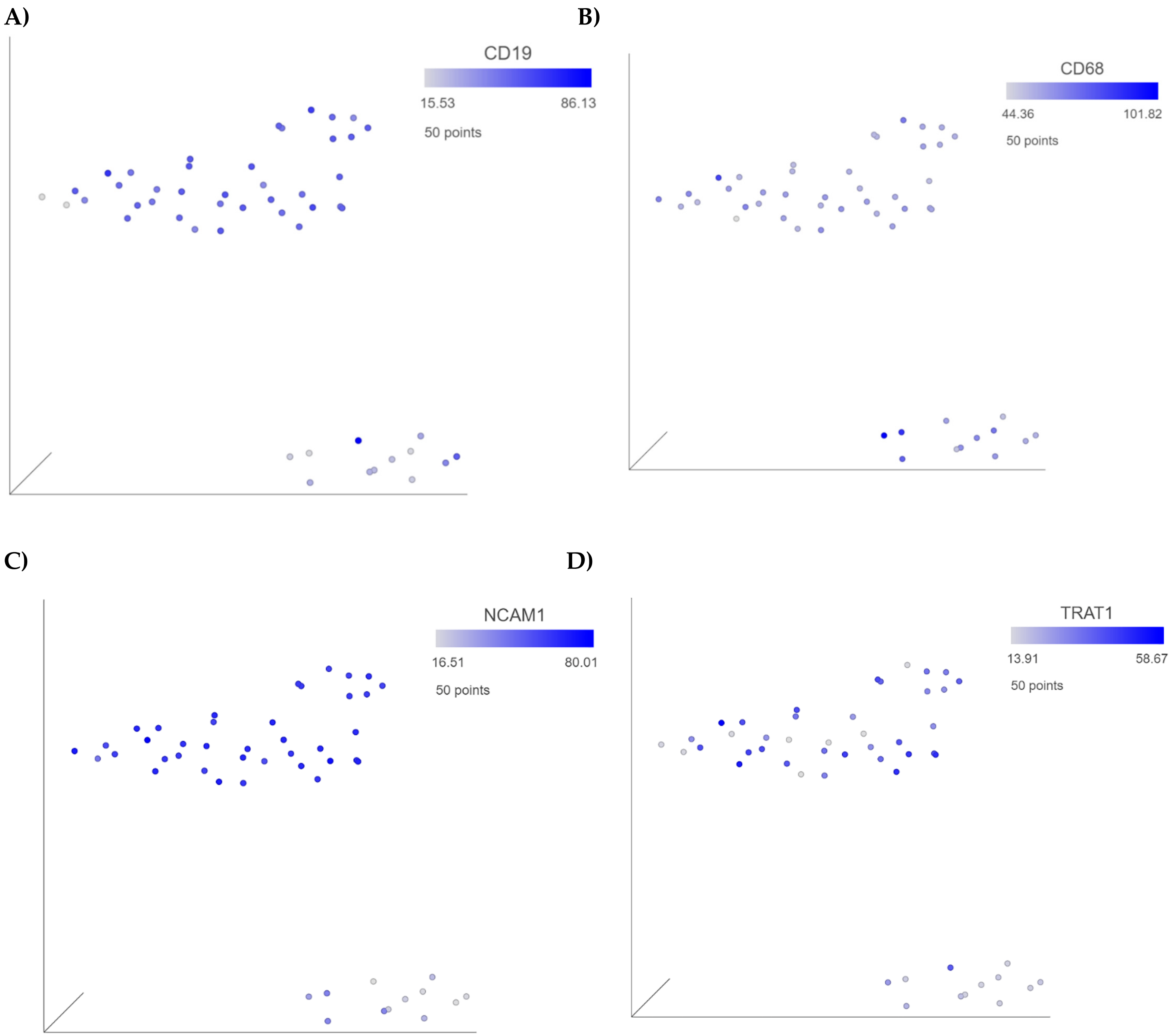

Figure S1. (**A**,**B**) B cell and macrophage expression in patients from dataset GSE162835. (**C**,**D**) Natural killer cell and T cell expression in patients from dataset GSE162835.

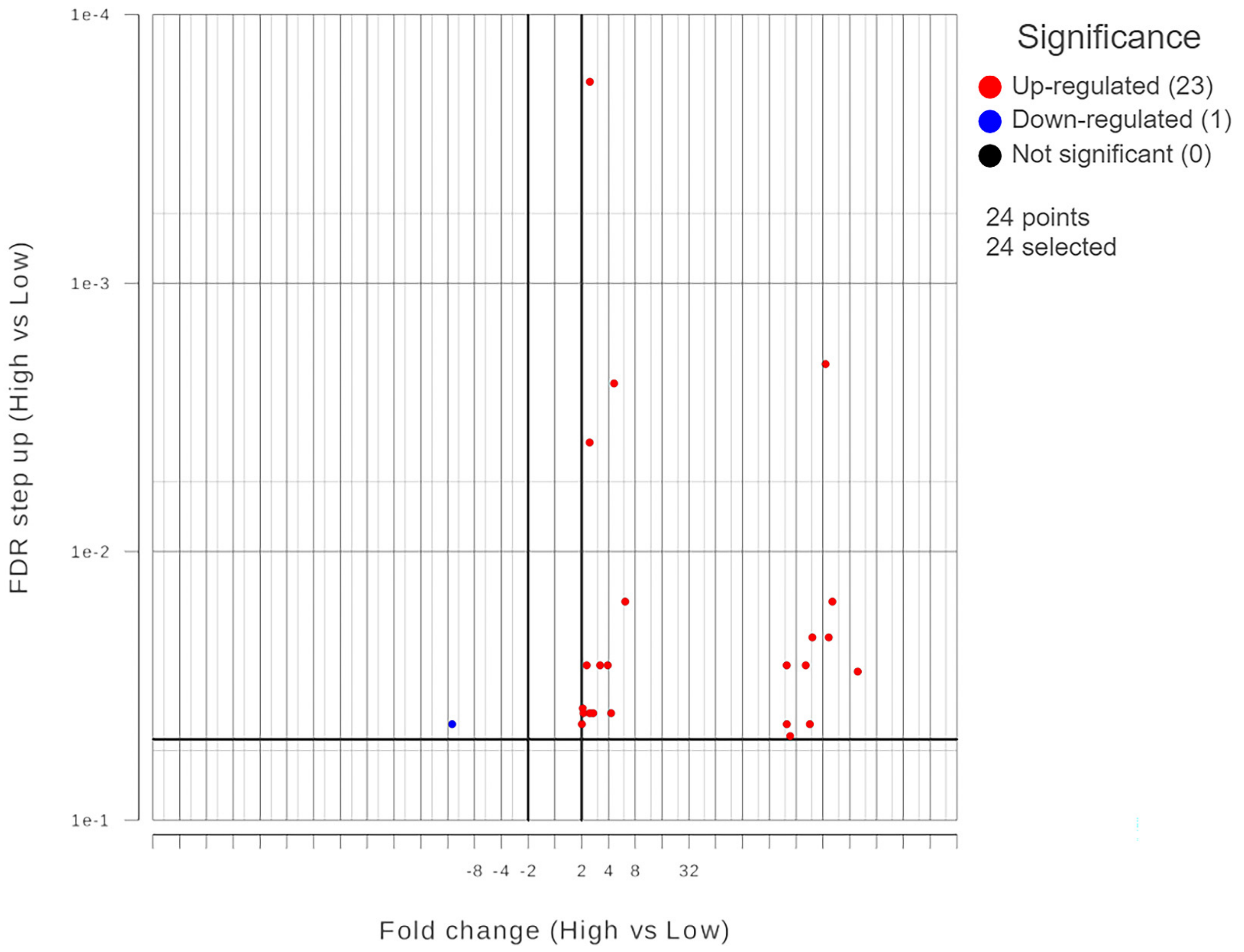

Figure S2. GSE150316 Volcano Plot of High versus Low Viral Load. Red dots are upregulated genes and blue dots are downregulated genes.

A) B)

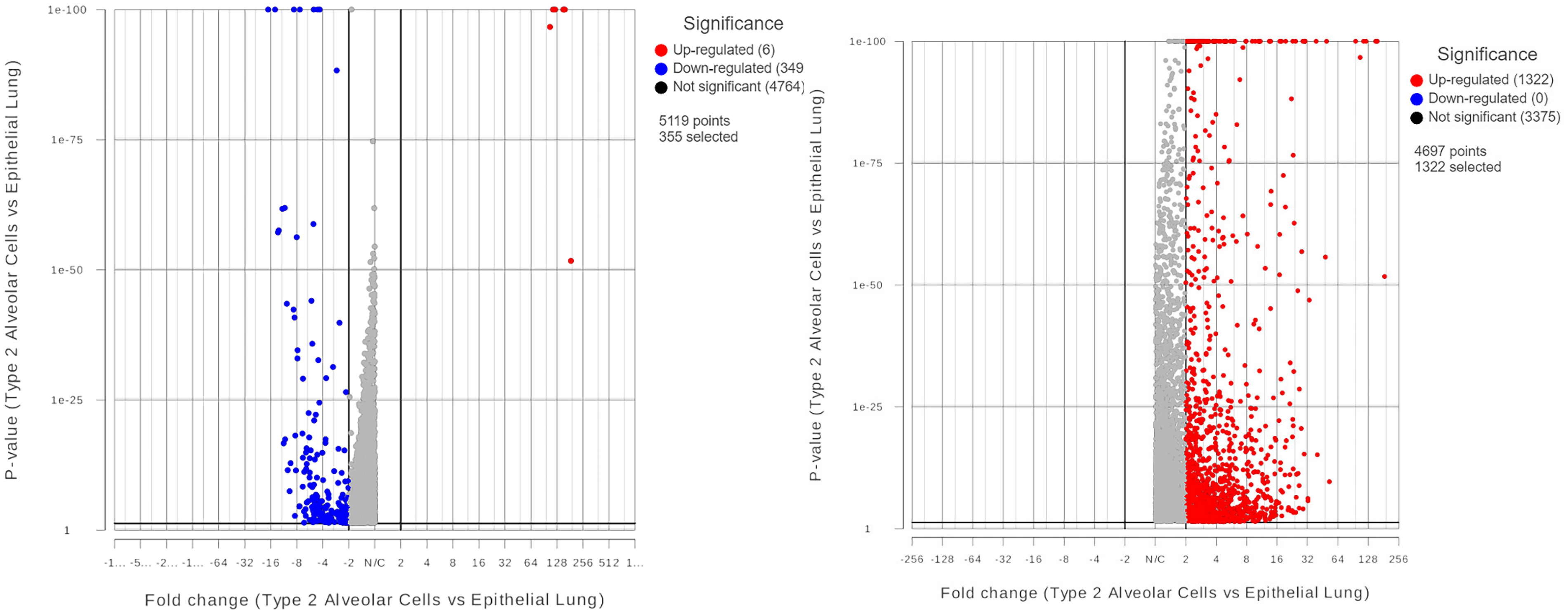

Figure S3. (**A**,**B**) GSE168215 Volcano Plots of AT2 Cells versus General Lung Epithelia. Red dots are upregulated genes and blue dots are downregulated genes.

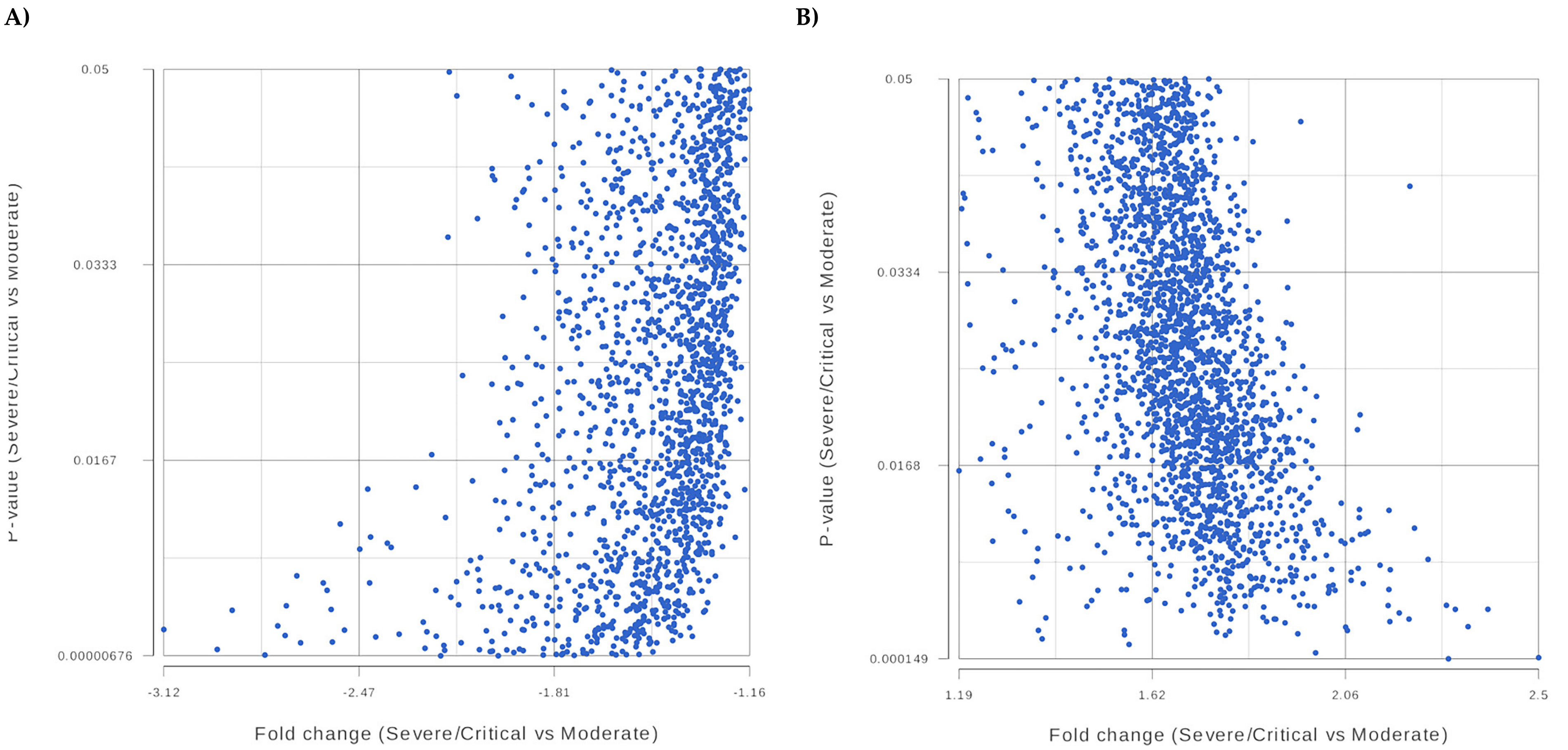

Figure S4. (**A**,**B**) Down- (left) and upregulated (right) genes comparing severe vs moderate patients from dataset GSE162835.

A)

B)

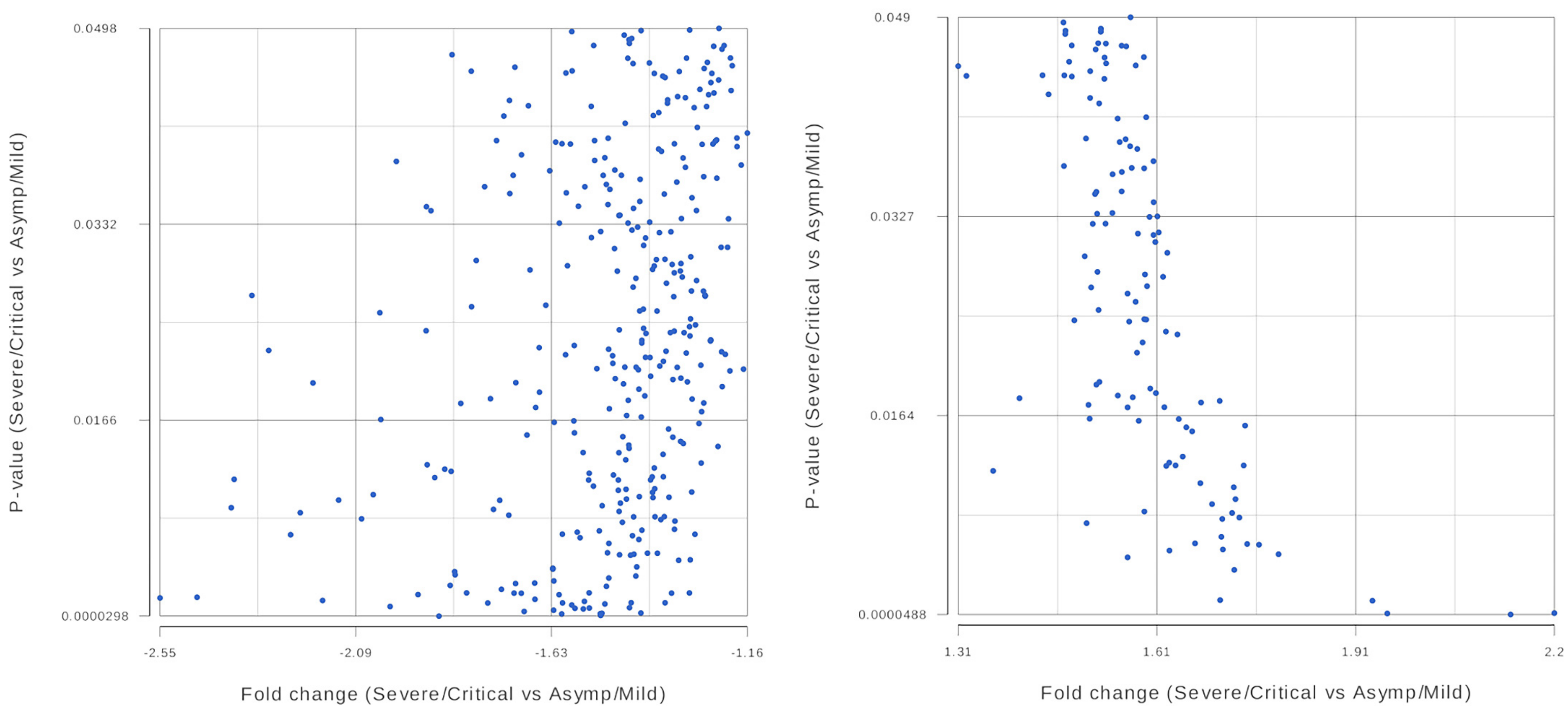

Figures S5. (**A**,**B**) Down- (left) and upregulated (right) genes comparing severe vs mild patients from dataset GSE162835.

A) B)

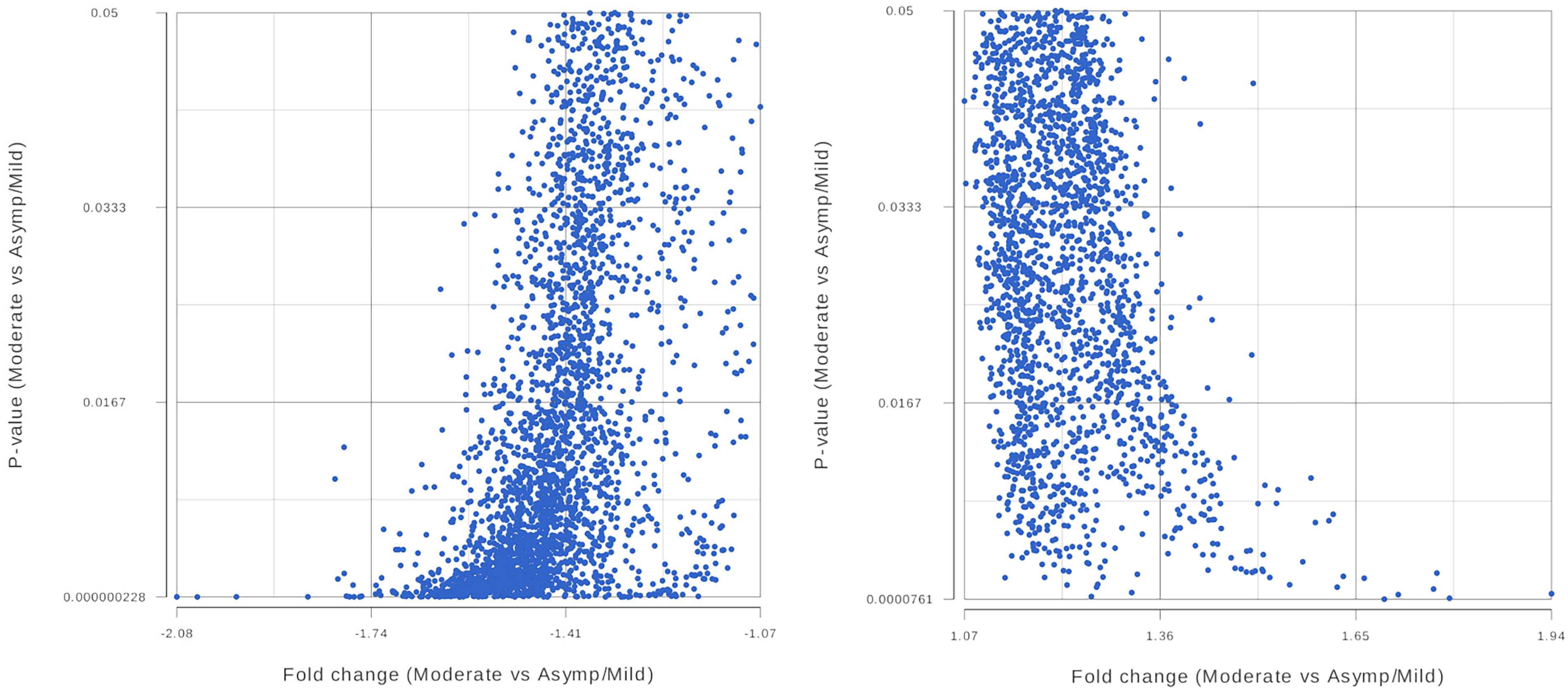

Figures S6. (**A**,**B**) Down- (left) and upregulated (right) genes comparing moderate vs mild patients from dataset GSE162835.

Host-Virus Interactions - Heart

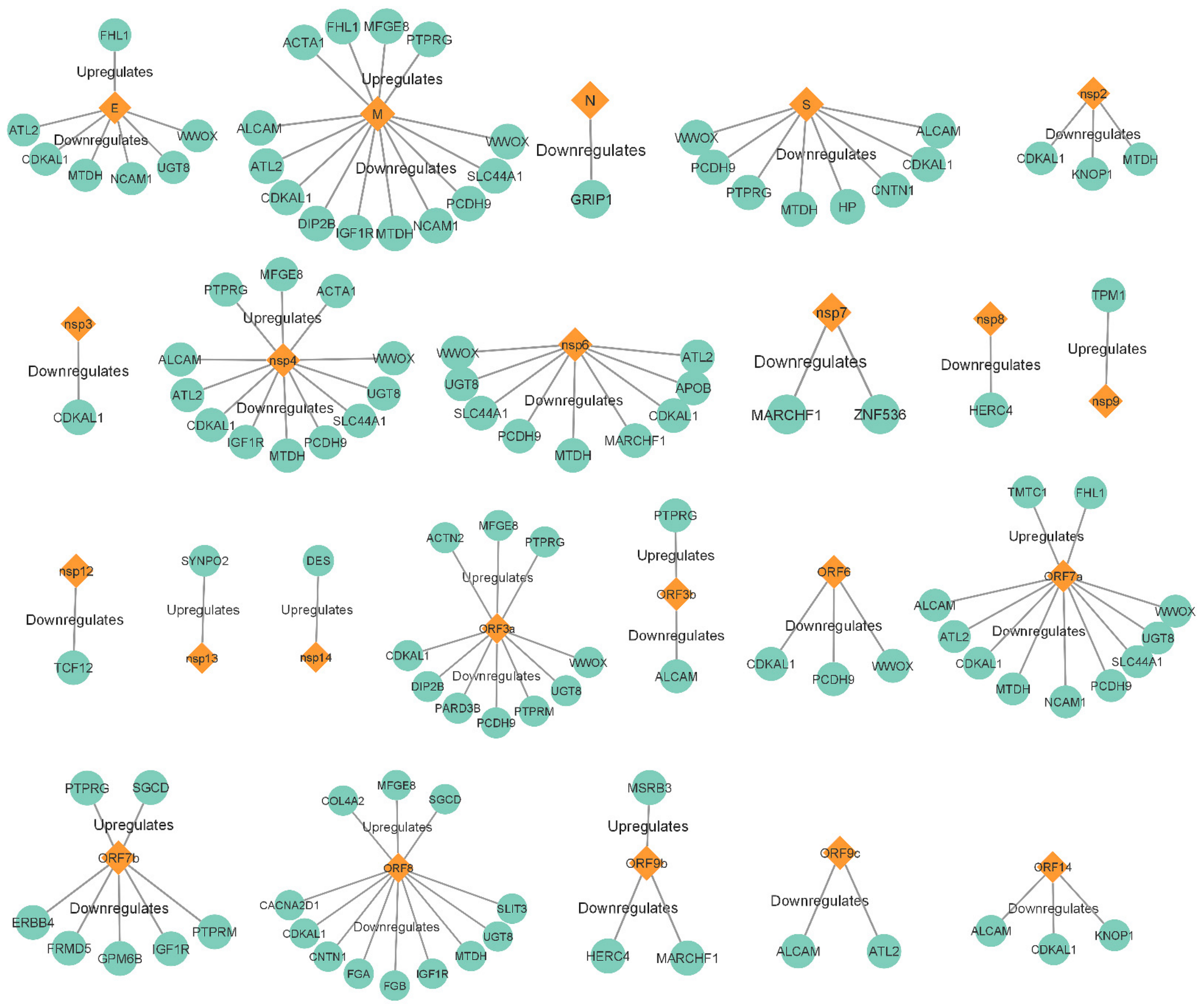

Figure S7. Viral-host protein mapping for heart samples. Interactions between SARS-CoV-2 proteins (orange diamonds) and human proteins (green circles). Interactions (black lines) were determined via comparison to BIOGRID's interaction database and were sorted by down- or upregulation of host proteins.

Host-Virus Interactions - Kidney

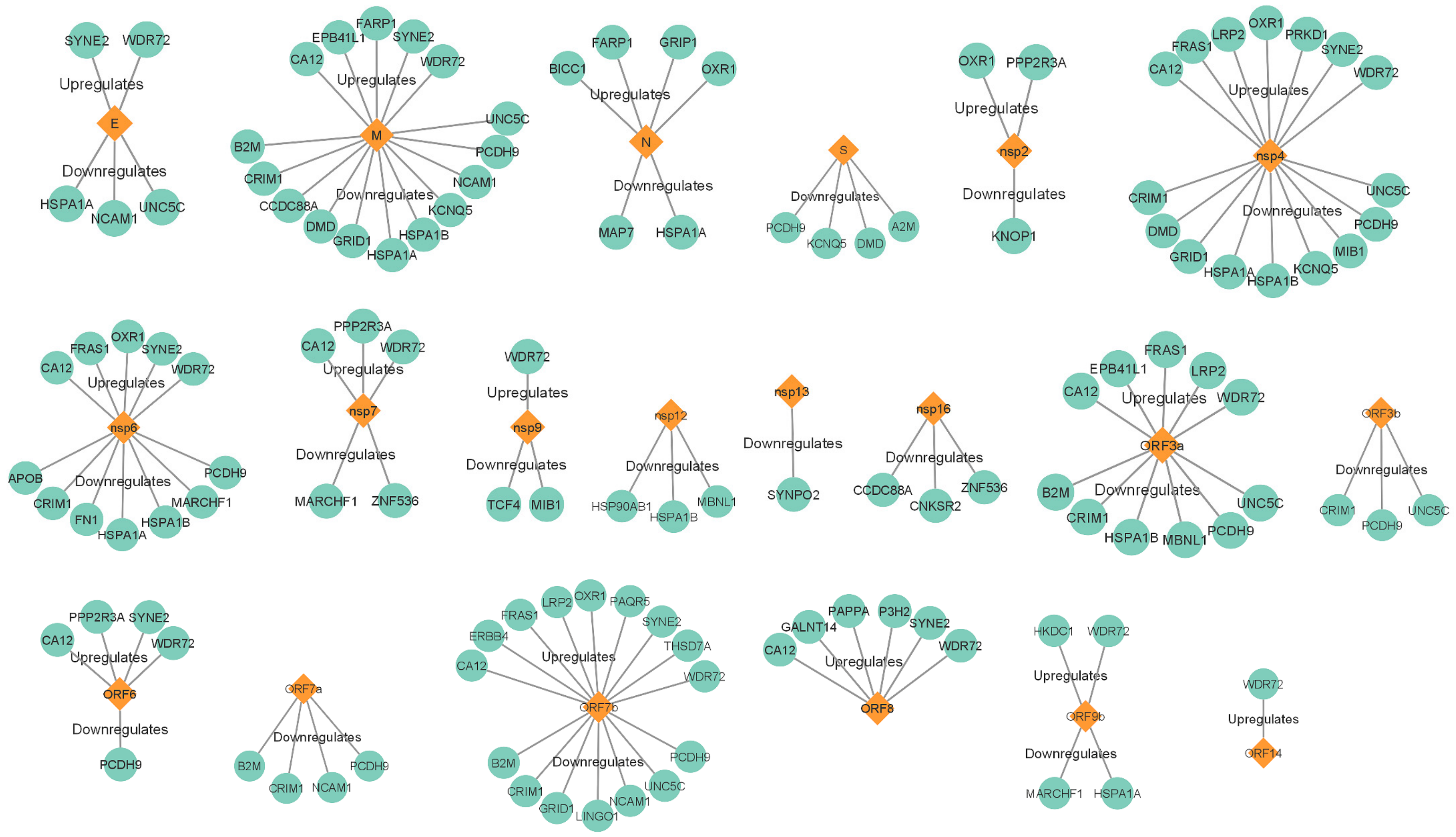

Figure S8. Viral-host protein mapping for kidney samples. Interactions between SARS-CoV-2 proteins (orange diamonds) and human proteins (green circles). Interactions (black lines) were determined via comparison to BIOGRID's interaction database and were sorted by down- or upregulation of host proteins.

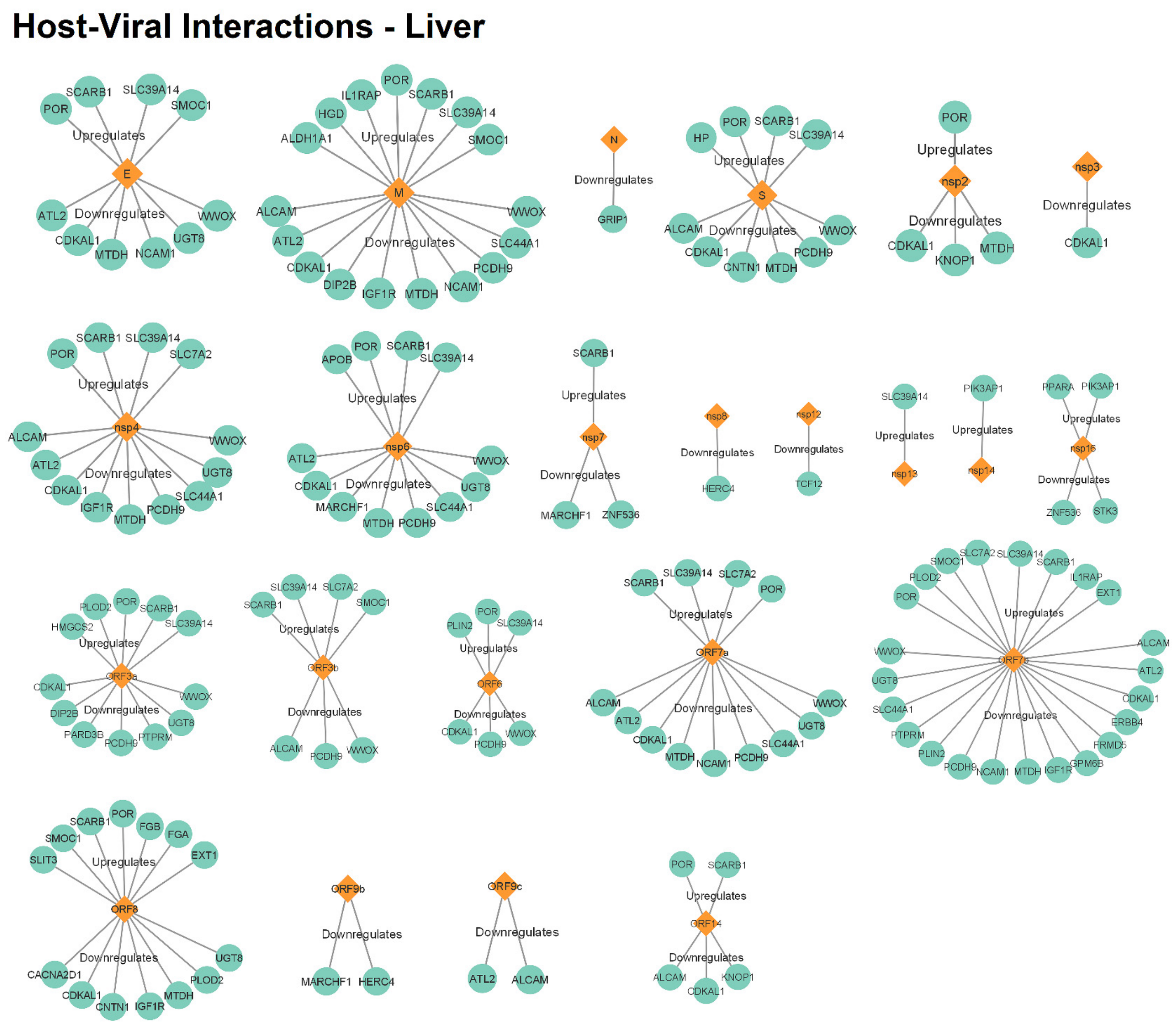

Figure S9. Viral-host protein mapping for liver samples. Interactions between SARS-CoV-2 proteins (orange diamonds) and human proteins (green circles). Interactions (black lines) were determined via comparison to BIOGRID's interaction database and were sorted by down- or upregulation of host proteins.

Host-Viral Interactions - Lung

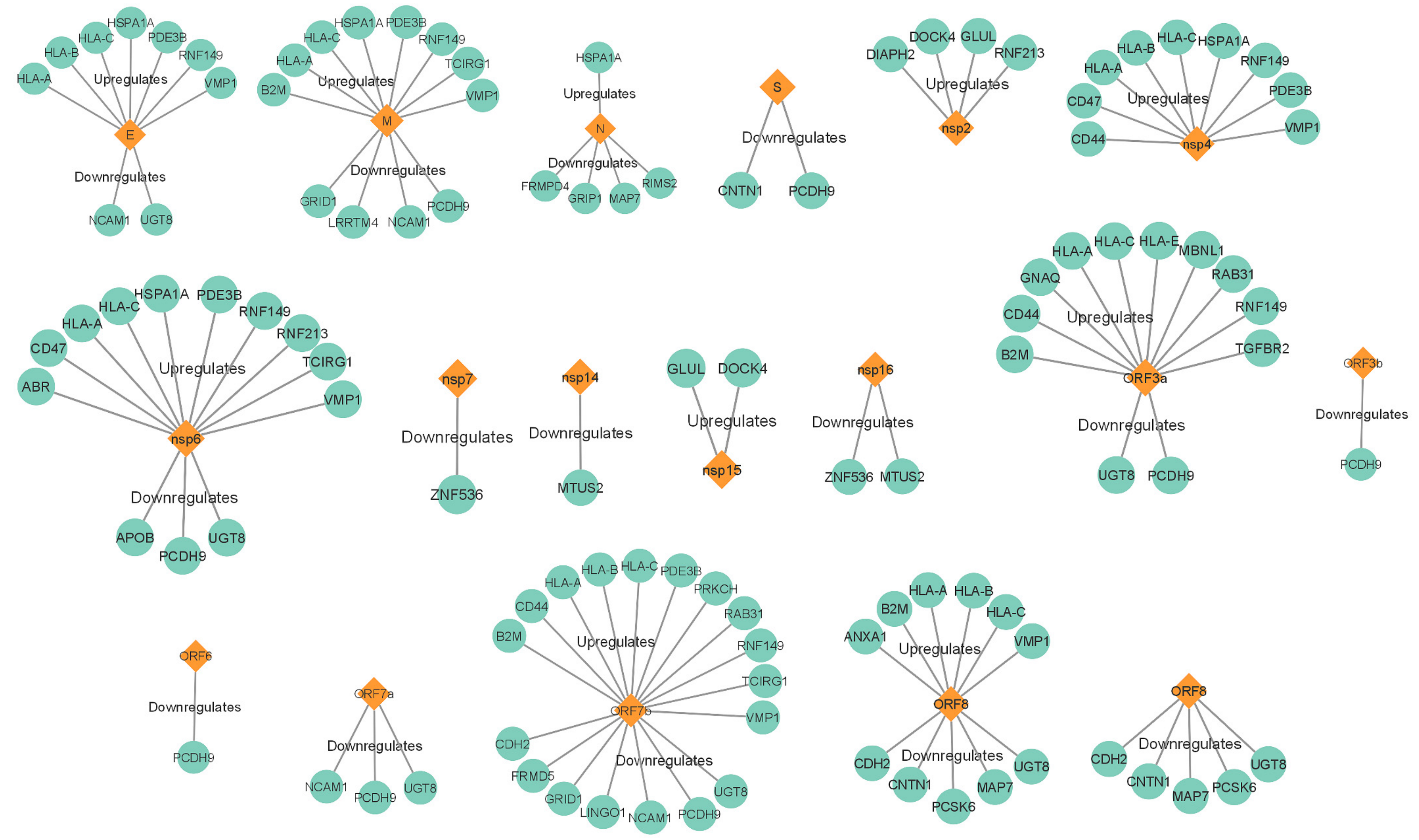

Figure S10. Viral-host protein mapping for lung samples. Interactions between SARS-CoV-2 proteins (orange diamonds) and human proteins (green circles). Interactions (black lines) were determined via comparison to BIOGRID's interaction database and were sorted by down- or upregulation of host proteins.
